# *Posidonia oceanica* Extract Inhibits VEGF-Induced Angiogenic and Oxidative Responses in Human Endothelial Colony-Forming Cells

**DOI:** 10.3390/jox15050153

**Published:** 2025-09-17

**Authors:** Francesca Margheri, Cecilia Anceschi, Elena Frediani, Alessandra Marzoppi, Marzia Vasarri, Donatella Degl’Innocenti, Emanuela Barletta, Anna Laurenzana, Anastasia Chillà

**Affiliations:** 1 Department of Experimental and Clinical Biomedical Sciences, University of Florence, Viale Morgagni 50, 50134 Florence, Italy; francesca.margheri@unifi.it (F.M.); cecilia.anceschi@unifi.it (C.A.); elena.frediani@unifi.it (E.F.); marzoppi.a@gmail.com (A.M.); marzia.vasarri@unifi.it (M.V.); donatella.deglinnocenti@unifi.it (D.D.); anna.laurenzana@unifi.it (A.L.); 2 Interuniversity Center of Marine Biology and Applied Ecology “G. Bacci” (CIBM), Viale N. Sauro 4, 57128 Leghorn, Italy

**Keywords:** angiogenesis, VEGF, marine drugs, *Posidonia oceanica*, antioxidant agents, endothelial cells

## Abstract

Angiogenesis, the formation of new blood vessels from pre-existing vasculature, is essential for physiological processes such as development and wound healing, but its dysregulation contributes to a range of pathological conditions including cancer, diabetic retinopathy, and chronic inflammation. In recent years, marine-derived compounds have emerged as promising multitarget agents with anti-angiogenic potential. *Posidonia oceanica*, a Mediterranean seagrass traditionally used in folk medicine, is increasingly recognized for its pharmacological properties, including antioxidant, anti-inflammatory, and anti-invasive activities. This study investigated the effects of a hydroethanolic extract from *P. oceanica* leaves (POE) on human Endothelial Colony-Forming Cells (ECFCs), a subpopulation of endothelial progenitor cells with high proliferative and vessel-forming capacity, and a relevant model for studying pathological angiogenesis. ECFCs were treated with POE (4–8 µg/mL), and cell viability, morphology, migration, invasion, tube formation, oxidative stress, and activation markers were evaluated. POE did not alter ECFC morphology or viability, as confirmed by Trypan Blue and MTT assays. However, functional assays revealed that POE significantly impaired ECFC migration, invasion, and in vitro angiogenesis in a dose-dependent manner. Under VEGF (Vascular endothelial growth factor) stimulation, POE reduced intracellular ROS accumulation and downregulated key redox-regulating genes (*hTRX1*, *hTRX2*, *PRDX2*, *AKR1C1*, *AKR1B10*). Western blot analysis showed that POE inhibited VEGF-induced phosphorylation of KDR, mTOR and p-ERK, while p-AKT remained elevated, indicating selective disruption of VEGF downstream signaling. Furthermore, POE reduced the expression of pro-inflammatory and pro-coagulant markers (*VCAM-1*, *ICAM-1*, *TF*) and partially reversed TNF-α–induced endothelial activation. These findings suggest that POE exerts anti-angiogenic effects through a multitargeted mechanism, supporting its potential as a natural therapeutic agent for diseases characterized by aberrant angiogenesis.

## 1. Introduction

Angiogenesis, the formation of new blood vessels from pre-existing vasculature, is essential for physiological processes such as embryonic development, wound healing, and tissue regeneration [1], but when deregulated it contributes to numerous pathological conditions [2]. These include cancer, diabetic retinopathy, wet age-related macular degeneration (AMD), rheumatoid arthritis, psoriasis, and chronic inflammatory diseases [3]. In these states, excessive or aberrant angiogenesis leads to tissue damage, leakage, fibrosis, and tumor progression, often worsening clinical outcomes.

Vascular endothelial growth factor A (VEGF A) binds to its receptor VEGFR2 (KDR) to trigger receptor phosphorylation and activate downstream pathways such as PI3K/AKT, mTOR, ERK, and reactive oxygen species (ROS) generation, which collectively drive endothelial proliferation, migration, and capillary morphogenesis [4]. ROS also act as signaling mediators enhancing VEGF’s effects, but excess ROS disrupt redox balance and promote endothelial dysfunction [5]. The interplay between angiogenesis, oxidative stress, and inflammation underscores the need for interventions that address all three components.

Clinically, anti-angiogenic therapies targeting VEGF signaling have become integral treatments for cancer and ocular vascular diseases [3,6]. Monoclonal antibodies like bevacizumab and ranibizumab, and tyrosine kinase inhibitors (sunitinib, sorafenib, nintedanib), are approved for various malignancies and neovascular AMD [7]. Despite initial efficacy, limitations include treatment resistance, off-target effects, rebound angiogenesis upon cessation, and high cost. Combination strategies, such as pairing anti angiogenic drugs with immune checkpoint inhibitors, have improved outcomes in renal cell carcinoma and lung cancer, yet resistance mechanisms like vascular mimicry and compensatory angiogenic pathways persist [8].

As such, research is advancing toward targeting multiple angiogenic cascades and exploring novel agents. For example, aptamers like pegaptanib, multitarget angiokinase inhibitors (e.g., nintedanib), and vascular disrupting agents are in development. Biologicals such as tumstatin peptides inhibit integrin mediated survival pathways, representing anti angiogenic strategies beyond direct VEGF blockade [9].

Concurrently, natural products, especially marine-derived compounds, are drawing attention due to their diverse chemical structures and bioactivities. Among these, algal carotenoids such as fucoxanthin, siphonaxanthin, and astaxanthin, isolated from brown and microalgae, have demonstrated to inhibit endothelial functions and VEGF signaling in cancer models. These bioactive pigments reduce oxidative stress, inhibit endothelial cell proliferation and migration, and interfere with key angiogenic signaling pathways, highlighting their potential as natural modulators of vascular growth [10]. In addition, sponge-derived compounds like Puupehenone, a marine-derived sesquiterpene isolated from sponges of the orders Verongida and Dictyoceratida, have been shown to suppress endothelial proliferation and differentiation interfering with vascular network formation, limiting neovascularization and tumor growth [11]. Fucoidan, a sulfated polysaccharide from brown seaweed, impairs endothelial proliferation and tube formation. In vivo, fucoidan enhances the effects of bevacizumab and sorafenib against hepatocellular carcinoma [12]. These findings illustrate the potential of marine phytocompounds in anti-angiogenic drug development [13,14].

*Posidonia oceanica*, a seagrass endemic to the Mediterranean Sea, is widely recognized for its ecological importance in marine environments. A first analysis conducted using UPLC revealed that POE has a predominantly polyphenolic content. The composition percentages are reported in Table 1.

In recent years, however, growing interest has emerged around its potential as a source of bioactive compounds with diverse potential pharmacological applications [15]. Preclinical studies increasingly support the potential health-promoting properties of *P. oceanica* leaves, which demonstrate antioxidant, anti-inflammatory, antidiabetic, and anti-glycation effects both in vitro and in vivo animal models [15,16,17,18,19,20]. Notably, POE has been shown to influence key cellular signaling pathways, such as ERK/AKT/mTOR, and to induce autophagy, thereby inhibiting tumor cell migration, invasion, and gelatinase activity [21,22]. Moreover, POE has been shown to reduce lipopolysaccharide (LPS)-induced reactive oxygen species (ROS) production and inflammatory responses in macrophages. The phytocomplex multitargeted mechanisms of action offer promising avenues for overcoming challenges such as drug resistance in cancer therapy, while its traditional use for managing conditions like diabetes and hypertension underscores its therapeutic relevance [16]. Emerging nanotechnology-based delivery systems further enhance its bioavailability, reinforcing the potential of *P. oceanica* as a valuable candidate for the development of novel treatments targeting inflammation, metabolic disorders, skin aging, and cancer [16]. By bridging centuries-old traditional applications with contemporary scientific research, *P. oceanica* stands as a compelling example of marine phytotherapy emerging role.

Given the overlapping molecular pathways involved in angiogenesis, inflammation, and oxidative stress, this study aimed to investigate the anti-angiogenic and anti-invasive effects of POE in Endothelial Colony-Forming Cells (ECFCs), a highly proliferative subpopulation of endothelial progenitor cells with strong vessel-forming potential. Due to their pivotal role in both physiological and pathological angiogenesis, ECFCs serve as a relevant in vitro model for studying endothelial function [23,24].

This study investigates the effects of POE (4–8 µg/mL of polyphenol equivalents) on human ECFC viability, morphology, migration, invasion, capillary-like tube formation, ROS generation, as well as redox- and inflammation-related gene expression. Additionally, we examined its impact on VEGF-driven signaling pathways (including KDR, AKT, ERK, mTOR). This study aims to evaluate whether POE can selectively modulate ECFC responses to VEGF, offering a promising avenue for targeting pathological angiogenesis.

## 2. Materials and Methods

### 2.1. Preparation of ECFCs and Cell Culture

Endothelial Colony-Forming Cells were isolated from >50 mL of human umbilical cord blood (UCB) obtained from healthy newborns, following maternal informed consent, as previously described [25]. Samples were collected at the Florence Cord Blood Bank. The expression of specific surface markers (CD45, CD34, CD31, CD105, ULEX, vWF, KDR, and uPAR) was assessed by flow cytometry. The isolation and use of stem cells derived from cord blood for research purposes is authorized under Italian legislation (Decree of 18 November 2009, Article 2, Paragraph 1, Letter f), contingent upon acquisition of maternal informed consent (Form R711-D). ECFCs were cultured in Endothelial Growth Medium-2 (EGM-2, Euroclone, Milano, Italy), supplemented with 10% (*v*/*v*) fetal bovine serum (FBS, Euroclone), and maintained at 37 °C in a humidified atmosphere with 5% CO_2_.

### 2.2. Preparation of P. oceanica Leaf Extract (POE)

The hydrophilic extract from *P. oceanica* leaves (POE) was prepared as previously described [21,22]. Briefly, dried and minced *P. oceanica* leaves were suspended overnight in 70% (*v*/*v*) EtOH (10 mL per gram of dry leaves) at room temperature under constant stirring. The mixture was subsequently heated for 3 h at 65 °C. After centrifugation at 2000× *g* to remove solid debris, the supernatant was mixed with n-hexane in a 1:1 ratio and vigorously shaken to separate hydrophobic constituents. The hydrophilic fraction (lower aqueous phase) was collected, aliquoted (1 mL), and dried using a Univapo™ vacuum-spin concentrator. For experimental use, a single batch of dried extract was reconstituted in 0.5 mL of 70% (*v*/*v*) EtOH and referred to as POE. Hydroethanolic extraction of 4 g of dried *P. oceanica* leaves (POE) yielded 5.2 mg of dry extract per aliquot, with a final concentration of 10.4 mg/mL (dry weight) upon resuspension in 0.5 mL of 70% EtOH. Given the high polyphenols of POE (approximately 88%) as previously reported [22], concentrations were expressed as polyphenols equivalents to better reflect the phenolic fraction responsible for the biological activity and to ensure consistency with previously published studies on *P. oceanica* extracts.

#### Determination of Total Polyphenol Content and Antioxidant Activity of POE

Total polyphenol content, antioxidant capacity, and radical scavenging activity were assessed using established colorimetric assays as previously reported [21,22]. TP content of POE was determined using the Folin–Ciocalteu colorimetric method [21], with gallic acid (0.5 mg/mL) as a standard over the range of 0–10 mg. TP values were expressed as milligrams of gallic acid equivalents per milliliter of reconstituted extract. Antioxidant capacity and radical-scavenging activity were evaluated using the ferric-reducing antioxidant power (FRAP) assay and the DPPH (α,α-diphenyl-β-picrylhydrazyl) assay, respectively [21], with ascorbic acid (0.1 mg/mL) used as a reference standard over the range of 0–4 mg. Results were expressed as milligrams of ascorbic acid equivalents per milliliter of extract.

### 2.3. Cellular Viability

Trypan blue staining: ECFCs were seeded in 6 cm culture dishes at a density of 2 × 10^5^ cells per dish and incubated in a humidified atmosphere containing 5% CO_2_. Cells were exposed to POE for 24 and 48 h at a final concentration of 4, 6 and 8 µg/mL (of polyphenol equivalents), in EGM2. Cytotoxicity was assessed using trypan blue exclusion assay. Briefly, 20 μL of cell suspension was mixed with an equal volume of 0.4% (*w*/*v*) trypan blue solution prepared in 0.81% NaCl and 0.06% (*w*/*v*) dibasic potassium phosphate. Viable (unstained) and non-viable (trypan blue-positive) cells were counted separately using a dual-chamber hemocytometer under a light microscope.

MTT assay: For the assay, 5000 cells per well were seeded in a 96-well plate using complete medium supplemented with 10% FBS, in a final volume of 100 µL. Three technical replicates were performed for each condition. The day after cell seeding, POE was added at various concentrations, diluted in medium containing 2% FBS, for 24 and 48 h. After treatment, the medium was removed and replaced with 100 µL of MTT solution (prepared from a 5 mg/mL stock solution and diluted 1:10 in DMEM without phenol red, to avoid interference with colorimetric quantification). The plate was incubated at 37 °C for approximately 1 h, until formazan crystals became visible. Following incubation, the MTT solution was removed and replaced with 100 µL of pure dimethyl sulfoxide (DMSO, Sigma Aldrich/Millipore, Burlington, MA, USA) to solubilize the formazan crystals.

The absorbance of the resulting formazan solution was measured at 595 nm using a microplate reader (Bio-Rad, Milan, Italy). The entire procedure was repeated for the additional plates at later time points.

### 2.4. Immunofluorescence Analysis

Rhodamine Phalloidin Reagent (ab235138, Abcam, Cambridge, United Kingdom) was applied to the cells to visualize cell morphology and the arrangement of actin cytoskeleton. DAPI were used for nucleus staining. The coverslips with the immune-labelled cells were mounted with an anti-fade mounting medium (Biomeda, Collegno, Italy) and analyzed under a Bio-Rad MRC 1024 ES confocal laser scanning microscope (Bio-Rad, Hercules, CA, USA) equipped with a 15 mW Krypton/Argon laser source. The cells were observed with a Nikon Plan Apo X60 oil immersion objective (Nikon Instruments, Rome, Italy) at 595 nm. Series of optical sections (X- and Y-steps: 512 × 512 pixels) were then obtained through the depth of the cells, with a thickness of 1 μm at intervals of 0.8 μm (Z-step). A single composite image was obtained by superimposition of 20 optical sections for each sample.

### 2.5. Scratch Assay

The scratch assay, also known as the wound healing assay, is a standardized method used to evaluate cell migration. Cells are seeded into 6-well plates and incubated until reaching the desired confluence (approximately 99%). Once confluence is achieved, a wound (scratch) is created in the cell monolayer using a sterile pipette tip. Following the scratch, the medium is aspirated and the wells are gently washed with PBS to remove cell debris generated by the scratch. PBS is then removed, and fresh EBM-2 medium supplemented with 2% FBS and POE at concentrations of 8, 12, and 16 μg/mL is added. Images of the scratch area are acquired at 4× magnification using a microscope (time zero). The plates are then incubated at 37 °C for 24 h, after which images of the same area are taken again to monitor wound closure. Data analysis is performed by measuring the scratch area at time zero and after 24 h using ImageJ 1.53s software.

### 2.6. Invasion Assay with Transwell Chambers

Cell invasion was assessed using Transwell chambers with 8 μm pore-size polycarbonate membranes (Corning, Corning, NY, USA), pre-coated with Geltrex (Gibco/Thermo Fisher Scientific, Waltham, MA, USA) to simulate the extracellular matrix. ECFCs were seeded in the upper chamber at a density of 25 × 10^3^ cells per well after POE treatment for 18 h. In the experiments with VEGF, it was added in the lower chamber as chemoattractant at the concentration of 50 ng/mL. The chambers were incubated at 37 °C in a humidified atmosphere with 5% CO_2_ for 6 h, a time frame compatible with typical mesenchymal-mode cell migration. Following incubation, membranes were fixed in methanol. Non-invading cells on the upper surface of the membrane were gently removed using a cotton swab, while cells that had migrated to the lower surface were stained with Crystal violet and quantified under a light microscope at 40× magnification in 10 randomly selected fields per membrane. Cell mobilization was determined by counting the number of cells that traversed the membrane. All experiments were performed in triplicate, and results were expressed as either the absolute number of migrated cells ± standard deviation (SD) or as a percentage relative to the control.

### 2.7. In Vitro Capillary Morphogenesis

In vitro capillary morphogenesis was performed as previously described [26] using tissue culture wells pre-coated with Geltrex (GIBCO). After POE treatment for 18 h, ECFCs were seeded at a density of 16 × 10^3^ cells per well in EGM-2 medium supplemented with 2% fetal bovine serum (FBS) and incubated at 37 °C in a humidified atmosphere containing 5% CO_2_. Images were captured at regular intervals using an EVOS optical microscope (Thermo Fisher Scientific, Monza, Italy). Quantitative analysis of capillary-like network formation was performed using the Angiogenesis Analyzer plugin for ImageJ software. In this analysis, “junctions” refer to structures composed of multiple nodes; “master junctions” were junctions linking at least three segments, they delimited the master segments; “meshes” are closed polygonal structures formed by multiple overlapping segments; and “segments” are linear elements bounded by two junctions.

### 2.8. ROS Detection Assay for Evaluating Oxidative Stress

The CellROX™ assay is a widely used fluorescence-based method for detecting and quantifying reactive oxygen species (ROS) in live cells. The probe we used was the CellROX™ Green (Thermo Fisher Scientific, Waltham, MA, USA) that operate via an oxidation-dependent mechanism: in their reduced form, the probes are non-fluorescent, but upon oxidation by ROS, they emit a detectable fluorescent signal.

For the assay, cells are seeded at a density of 9000 cells per well in a 96-well plate, in complete medium containing 10% FBS (200 μL per well). After approximately 6 h, once cell adhesion is achieved, cells are treated with various concentrations of POE diluted in medium containing 2% FBS. The following morning, the medium is aspirated and the CellROX™ probe solution is added.

The probe solution is prepared by diluting CellROX™ 1:500 in phenol red-free medium. A volume of 70 μL of this solution is added to each well, and the plate is incubated at 37 °C for 15 min. Fluorescence is then measured using a plate reader (BioTek Instruments, Winooski, VT, USA) at an excitation/emission wavelength of 488/520 nm. The results are normalized to cell viability, assessed via the MTT assay.

### 2.9. RNA Extraction, Quantitative PCR

Total RNA was extracted using Trizol reagent (Sigma-Aldrich), and RNA integrity was assessed by agarose gel electrophoresis. Complementary DNA (cDNA) was synthesized using a cDNA synthesis kit (Bio-Rad) according to the manufacturer’s protocol. Gene expression analysis was performed by quantitative real-time PCR (qRT-PCR) using the SsoAdvanced Universal SYBR Green Supermix (Bio-Rad) on a 7500 Fast Real-Time PCR System (Applied Biosystems, Waltham, MA, USA). Relative gene expression levels were calculated using the comparative Ct (ΔΔCt) method, with 18S rRNA serving as the reference gene. Amplification was carried out under the following cycling conditions: 95 °C for 10 s, followed by 40 cycles of 60 °C for 30 s. Primer sequences (IDT, TemaRicerca, Bologna, Italy) were as indicated in Table 2.

### 2.10. Western Blot Analysis

Harvested cells were lysed in RIPA buffer (pH 7.4; Merck Millipore, Vimodrone, MI, Italy) supplemented with a protease inhibitor cocktail (Calbiochem, Merck, Darmstadt, Germany), followed by sonication using a Microson XL-2000 sonicator (Misonix, Farmingdale, NY, USA). Equal amounts of protein (50 μg per sample), quantified and mixed with Laemmli buffer, were separated by SDS-PAGE using Bolt Bis-Tris Plus 4–12% precast polyacrylamide gels (Life Technologies, Monza, Italy). Proteins were then transferred onto a PVDF membrane using the iBlot 2 Dry Blotting System (Life Technologies, Monza, Italy). Membranes were briefly stained with Ponceau S solution to verify uniform protein loading and efficient transfer, then blocked for 1 h at room temperature with 6% (*w*/*v*) skimmed milk in PBS containing 0.1% Tween-20 (PBST). Following blocking, membranes were incubated overnight at 4 °C with specific primary antibodies: p-KDR/pVEGFR2 (Rabbit polyclonal 1:1000 Cell Signaling, Cat#2478), KDR/VEGFR2 (Rabbit polyclonal 1:1000 Cell Signaling, Cat#9698), p-AKT and AKT (rabbit, polyclonal 1:1000, Cell signaling Technology, p-AKT Cat#9271; AKT Cat#9272), GAPDH (rabbit polyclonal 1:1000, Cell signaling Technology, Cat# 2118), p-ERK (Phospho-p44/42 MAPK (Erk1/2) (Thr202/Tyr204) (rabbit polyclonal 1:1000 Cell signaling Technology, Cat#9101) and ERK2 (rabbit polyclonal 1:500 Santa Cruz Biotechnology, Cat#sc-154), p-mTOR and mTOR (Rabbit polyclonal 1:1000 Cell Signaling, p-mTOR Cat#2971 and mTOR Cat#2972); used to assess equal amounts of protein loaded in each lane. Antirabbit IgG (whole molecule)–Peroxidase antibody (1:5000 Cell Signaling, Cat#7074P2, Lot#33) was used as secondary antibodies; the enhanced chemiluminescence (ECL) procedure was employed for development.

### 2.11. Statistical Analysis

Data were analysed using GraphPad Prism6 and Origin and expressed as a mean value ± SD. Statistical analysis was performed using One-way Anova.

## 3. Results

### 3.1. Effect of POE on Endothelial Colony-Forming Cells (ECFC) Viability and Cell Morphology

Hydroethanolic extraction of 4 g of dried *P. oceanica* leaves (POE) yielded 5.2 mg of dry extract per aliquot, with a final concentration of 10.4 mg/mL (dry weight) upon resuspension in 0.5 mL of 70% EtOH. POE showed a total polyphenol content of 0.5 ± 0.06 mg/mL gallic acid equivalents, and demonstrated antioxidant and radical-scavenging activities equivalent to 0.097 ± 0.01 mg/mL and 0.62 ± 0.03 mg/mL of ascorbic acid equivalents, respectively.

**Figure 1:**
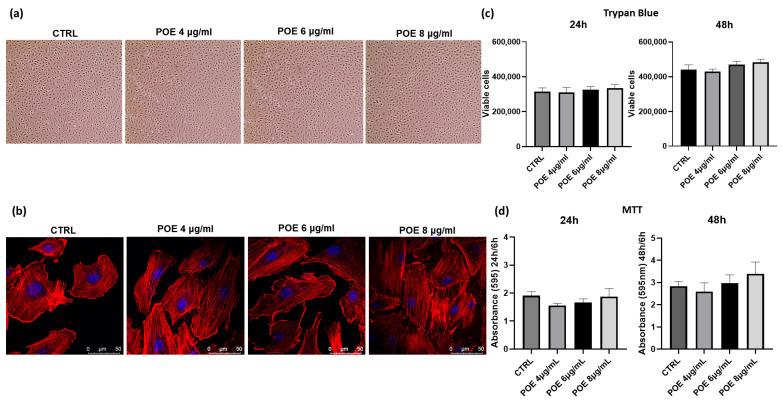
Effect of POE on ECFC morphology and viability. (**a**) ECFC morphology was observed using an optical microscope in cells treated with the EtOH vehicle and POE at concentrations of 4, 6 and 8 μg/mL. (**b**) Confocal microscopy was used to observe the morphological features of phalloidin-stained treated cells compared to the CTRL. Red: phalloidin staining of the actin cytoskeleton. Blue: nuclear staining with DAPI. Magnification 40 X. (**c**,**d**) ECFC viability after 24 and 48 h upon treatment with different concentration of POE evaluated by Trypan blue dye exclusion (**c**) and MTT assays (**d**). Values are shown in viable cell number for the Trypan blue and Absorbance (595 nm) for the MTT. In both assays POE treated cells were compared to vehicle-treated cells (CTRL). Values are plotted as mean ± SD of three independent experiments.

To assess the biological effects of POE, its impact on cell morphology and viability was evaluated in ECFCs following overnight treatment at varying concentrations. Morphological analysis was performed using both optical and confocal microscopy, as shown in Figure 1a,b. At 18 h post-treatment, control and POE-treated cells (4, 6, and 8 µg/mL of polyphenol equivalents) were imaged using a light microscope. For detailed morphological changes, cells were stained with rhodamine-phalloidin to selectively label F-actin. As shown in Figure 1a,b, no significant morphological alterations were observed in ECFCs across the tested POE concentrations, indicating that POE does not adversely affect cell morphology under these experimental conditions. Cell viability was assessed using the Trypan Blue exclusion assay after 24 and 48 h of treatment with increasing concentrations of POE (4 µg/mL, 6 µg/mL, 8 µg/mL). Cells treated with 70% EtOH vehicle were used as controls (CTRL) (Figure 1c). As shown in Figure 1a–c, no significant changes in cell morphology or cell viability were noted after POE treatment. Moreover, the results were corroborated by the MTT assay (Figure 1d) conducted at the same time points. The MTT assay revealed no toxicity at any of the POE concentrations used, and even the higher concentrations of POE showed a slight positive effect on cell proliferation at 48 h, although this effect was not statistically significant.

**Figure 2:**
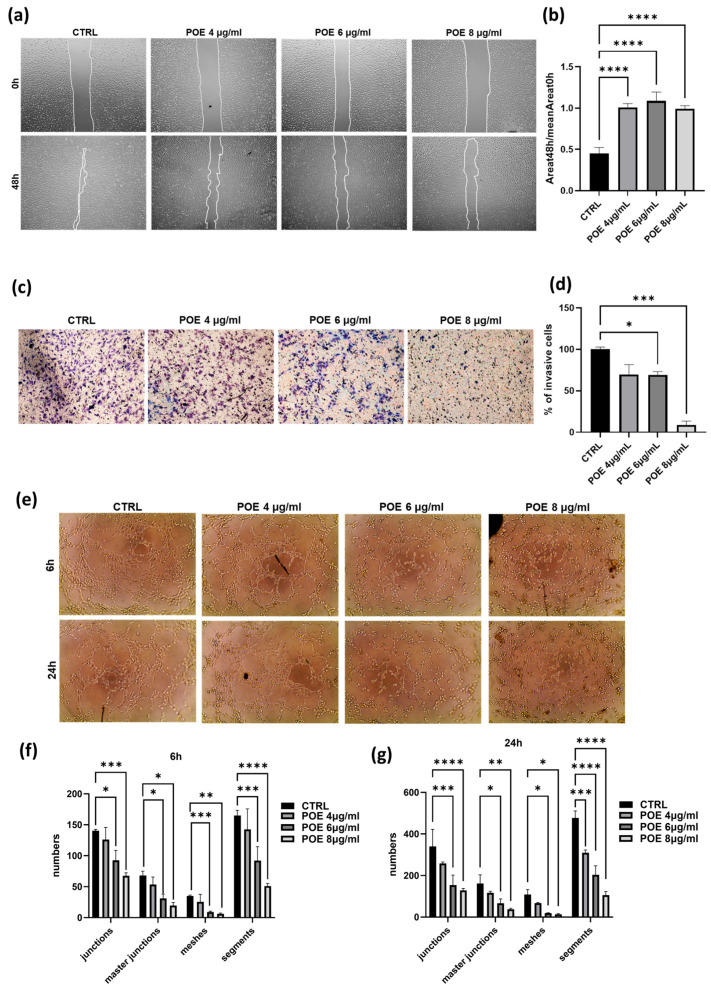
Effect of POE on ECFC migration, invasion and vessel formation. (**a**) Wound healing assay on ECFC treated with 70% EtOH (CTRL) or treated with POE (4, 6 and 8 μg/mL). Scratch closure was photographed after 48 h. White lines mark the edges of the wound area. (**b**) Analysis of scratch closure in POE-treated cells or control cells. Wound closure values were measured by considering the ratio of the wound area at 48 h to the wound area at 0 h after migration at different time points. (**c**) Representative image of ECFC invasion using Transwell chambers with Geltrex coated filters after POE treatments with different concentrations. Violet invasive cells shown in the pictures were stained with Crystal violet. Histogram in (**d**) refers to quantification of invasion assay obtained by counting the total number of invasive cells/filter. (**e**–**g**) Capillary morphogenesis of ECFCs treated with the vehicle or POE at different concentrations. Representative microphotographs (×10) of capillary-like structures at 6 and 24 h are shown (**e**). The capillary network was quantified by Angiogenesis Analyzer Image J tool. Histograms represent the mean of number of junctions, master junctions, meshes and segments ((**f**) at 6 h and (**g**) at 24 h). In this figure values are represented as means ± SD from three different experiments. * *p* < 0.05; ** *p* < 0.01; *** *p* < 0.001; **** *p* < 0.0001.

Different treatment durations were used to distinguish between early functional responses and later effects on cell proliferation and metabolic activity. A short 18 h treatment was applied for morphological assessment and functional assays (invasion and tube formation showed in Figure 2) to capture early cellular responses to POE exposure, before any changes in proliferation could influence the observed behavior. In contrast, longer treatments of 24 and 48 h were used in MTT and Trypan Blue assays to monitor potential cumulative effects on cell viability and proliferation over time. The results observed confirm the absence of toxicity of POE, as previously reported in the literature [21,22]. This validates the safety of the POE phytocomplex and highlights the importance of these findings for optimizing conditions in future therapeutic applications.

### 3.2. Effect of POE on ECFC Migration, Invasion and Angiogenic Capacity

The inhibitory effect of POE on cell migration was evaluated by wound healing assay. The vehicle control (CTRL) consisted of the same final % *v*/*v* ethanol as in the treatments (corresponding to the highest concentration used), but without POE (0 µg/mL). Wound area was analyzed using a phase-contrast microscope, and images were acquired at different time points, 0 and 48 h post-scratch. The ratio of the wound area at 48 h to the wound area at 0 h was calculated to determine the extent of closure (histogram in Figure 2b). As shown in Figure 2a,b, control cells were able to nearly complete the wound closure process, whereas cells treated with POE at all three concentrations showed significantly reduced migratory capacity, confirming the ability of POE to inhibit cell migration.

To further assess the impact of POE on ECFC invasion ability, a Transwell Assay was performed. Cells pre-treated for 18 h with 4 µg/mL, 6 µg/mL, and 8 µg/mL of POE were seeded onto wells pre-coated with Geltrex. The results presented in Figure 2c, indicate a dose-dependent reduction in the invasiveness of ECFCs treated with POE. 

These findings provide strong evidence of the inhibitory effect of POE on the invasive capacity of ECFC, in addition to the migratory effects described above.

Given the pronounced angiogenic potential of ECFC, we assessed their in vitro ability to form capillary-like tubular structures following treatment with POE. After POE treatment for 18 h, cells were seeded onto a three-dimensional Geltrex matrix, and phase-contrast images were acquired at 6 and 24 h post-treatment. These images were then analyzed using ImageJ software. As shown in Figure 2e–g, treatment with POE at the higher concentrations of 6 µg/mL and 8 µg/mL significantly inhibited the cells’ ability to organize into tubular structures resembling blood vessels, in a dose-dependent manner. In contrast, control cells were able to form tubular structures. These results indicate a potential inhibitory effect of POE on the angiogenic capacity of endothelial cells, suggesting its possible application as an anti-angiogenic agent.

### 3.3. Antioxidant Properties of POE in VEGF-Stimulated ECFCs

Reactive oxygen species (ROS) are key signaling molecules involved in the regulation of various physiological and pathological processes, including angiogenesis. In endothelial cells, controlled ROS production is essential for VEGF-induced functions such as proliferation, migration, and tubulogenesis. VEGF stimulation increases intracellular ROS levels primarily through activation of NADPH oxidases and mitochondrial pathways. While moderate ROS levels serve as signaling mediators, excessive ROS accumulation disrupts redox homeostasis, promoting oxidative stress, endothelial dysfunction, and aberrant angiogenesis [4,5,27]. Therefore, maintaining ROS balance is critical for proper endothelial function. In this context, natural compounds with antioxidant properties have gained increasing interest for their potential to modulate redox signaling in vascular biology. Notably, POE, previously shown to inhibit key angiogenic behaviors of ECFCs, may also exert a protective antioxidant effect. To explore this hypothesis, we assessed whether POE modulates intracellular ROS levels in ECFCs exposed to VEGF-induced oxidative stress.

Based on the previous results demonstrating a dose-dependent inhibition of ECFC angiogenic functions, we selected 8 µg/mL POE as the most effective concentration for subsequent experiments. This dose consistently showed the strongest inhibitory effects on migration, invasion, and tube formation without affecting cell viability or morphology. Therefore, all following assays involving VEGF stimulation were conducted using POE at 8 µg/mL to evaluate its potential to counteract VEGF-induced pro-angiogenic and oxidative responses.

**Figure 3:**
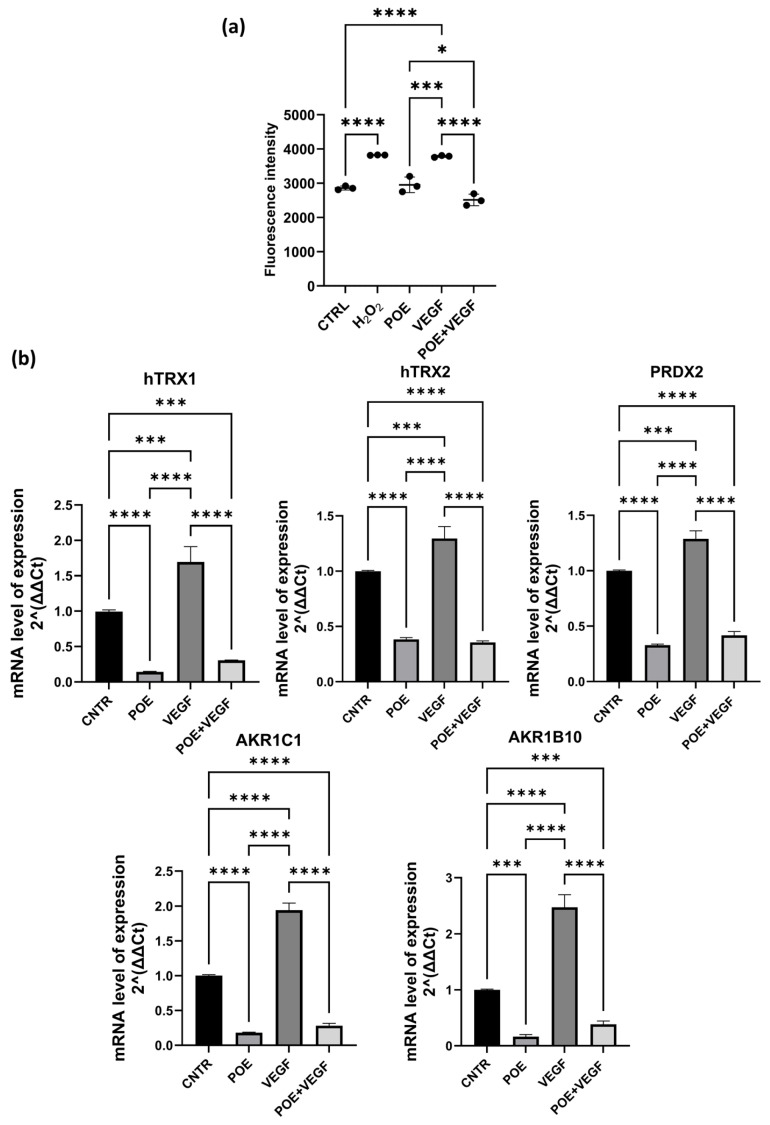
ROS levels and redox-related gene expression in POE treated-ECFC after VEGF stimulation. (**a**) CellROX fluorescence intensity at 488/520 nm after 18 h POE treatment measured using a plate reader for intracellular ROS quantification. Hydrogen peroxide (H_2_O_2_), a well-characterized inducer of oxidative responses, was employed as a positive control. VEGF was used at the concentration of 50ng/ml and POE at 8 μg/ml. (**b**) Relative mRNA expression levels of *hTRX1*, *hTRX2*, *PRDX2*, *AKR1C1*, and *AKR1B10* were analyzed by quantitative real-time PCR in ECFCs under different treatment conditions: control, POE alone (8 μg/mL), VEGF (50 ng/mL), and VEGF combined with POE. Gene expression levels were normalized to 18S and expressed as fold change relative to control. In this figure data are presented as mean ± SD of at least three independent experiments. Statistical significance was assessed by one-way ANOVA. * *p* < 0.05; *** *p* < 0.001; **** *p* < 0.0001.

Cells were stimulated with VEGF alone and in the presence of 8 µg/mL POE and intracellular ROS levels were quantified using the CellROX fluorescent assay. Hydrogen peroxide (H_2_O_2_), a well-characterized inducer of oxidative responses, was employed as a positive control. Data revealed that POE effectively mitigated the pro-oxidant effects elicited by VEGF stimulation suggesting a potential antioxidant role of POE in modulating redox homeostasis in ECFCs (Figure 3a).

Quantitative PCR analysis revealed that treatment with POE led to a downregulation of genes involved in redox regulation and detoxification, including *hTRX1*, *hTRX2*, *PRDX2*, *AKR1C1*, and *AKR1B10*. These genes play important roles in maintaining endothelial redox balance and modulating responses to oxidative stress. Specifically, *hTRX1* and *hTRX2* (thioredoxins 1 and 2) are redox-active proteins that participate in the reduction in disulfide bonds in cytoplasmic and mitochondrial proteins, respectively, and are critical for protecting endothelial cells from oxidative damage. *PRDX2* (peroxiredoxin 2) acts as a scavenger of hydrogen peroxide and helps regulate intracellular ROS levels during angiogenic signaling. *AKR1C1* and *AKR1B10* belong to the aldo-keto reductase family and are involved in detoxifying reactive carbonyl species generated under oxidative stress, contributing to cellular defense mechanisms in vascular cells.

Data shown in Figure 3b revealed that upon stimulation with VEGF, a known inducer of oxidative stress and angiogenesis, the expression of all five genes was significantly upregulated, reflecting the activation of endogenous antioxidant pathways in response to elevated ROS levels. Interestingly, co-treatment with POE and VEGF significantly attenuated the VEGF-induced overexpression of these genes, reducing their transcript levels closer to or below baseline. These results suggest that POE can mitigate VEGF-induced oxidative stress in ECFCs, potentially by lowering ROS levels and thus reducing the cellular need to activate redox-protective gene expression. This further supports a functional antioxidant role of POE in modulating redox homeostasis during angiogenic activation.

### 3.4. Anti-Invasive and Anti-Angiogenic Properties of POE in VEGF-Stimulated ECFCs

**Figure 4:**
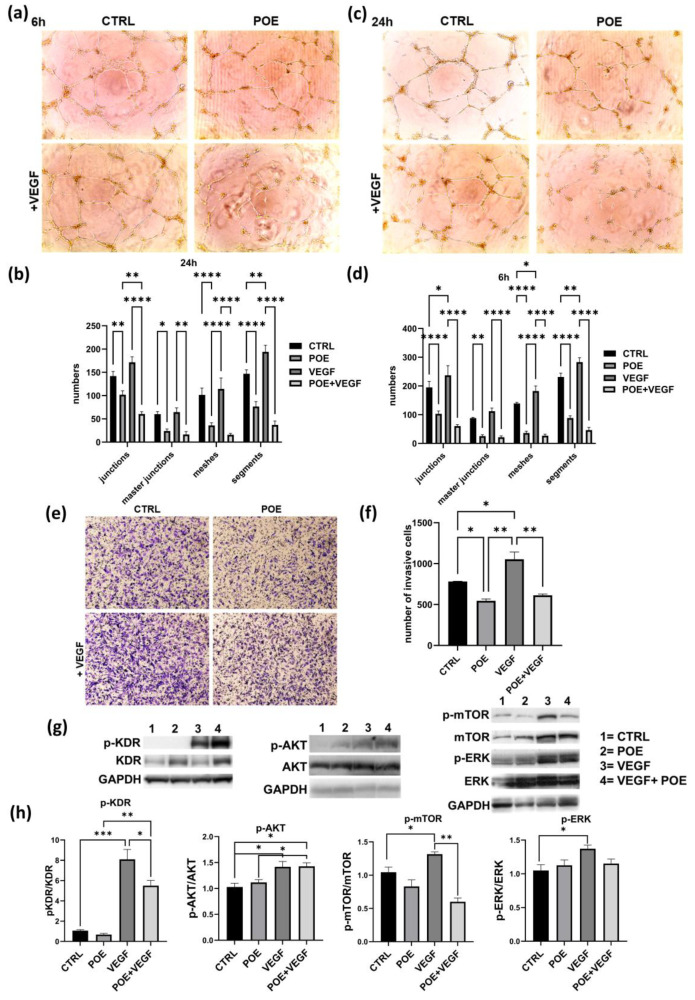
Effect of POE in VEGF-stimulated ECFCs on invasion and angiogenesis. (**a**) Capillary morphogenesis of ECFCs treated with the vehicle, VEGF (50 ng/mL) or POE (8 μg/mL) alone, or with the combination of VEGF and POE. Representative microphotographs (×10) of capillary-like structures at 6 h (**a**) and 24 h (**c**) are shown. The capillary network was quantified by Angiogenesis Analyzer Image J tool. Histograms represent the mean of number of junctions, master junctions, meshes and segments ((**b**) at 6 h and (**d**) at 24 h). (**e**) Representative image of ECFC invasion using Transwell chambers with Geltrex coated filters after VEGF (50 ng/mL) or POE (8 μg/mL) alone, or with the combination of VEGF and POE. Violet invasive cells shown in the pictures were stained with Crystal violet. Histogram in (**f**) refers to quantification of invasion assay obtained by counting the total number of invasive cells/filter. (**g**) Western blot analysis of important angiogenic cues (KDR, p-AKT, p-ERK and mTOR) in ECFCs treated with VEGF (50 ng/mL) or POE (8 μg/mL) alone, or with the combination of VEGF and POE. GAPDH is reported as loading control. (**h**) Histograms report quantification of signals from a densitometry analysis of at least three independent experiments. Results are the mean of 3 different experiments performed in duplicate. In this figure values are represented as means ± SD from three different experiments. * *p* < 0.05; ** *p* < 0.01; *** *p* < 0.001; **** *p* < 0.0001.

Building on the observation that POE modulates redox gene expression and mitigates VEGF-induced oxidative stress, we next sought to determine whether it could also counteract the pro-angiogenic effects of the main pro-angiogenic factor VEGF on ECFCs. As shown in the images obtained through the capillary morphogenesis assay (Figure 4a–d), VEGF treatment alone stimulated the formation of tubular structures, confirming its pro-angiogenic effect. When POE was combined with VEGF at 8 µg/mL, a clear anti-angiogenic and inhibitory effect of POE on ECFCs was observed, despite the presence of VEGF, as indicated by the histograms after the analysis at both 6 h (Figure 4b) and 24 h (Figure 4d).

A chemoinvasion assay was performed to investigate if POE could be able to interfere even with the VEGF mediated chemotactic action on ECFC invasion. As shown in Figure 4e and in the related histogram (Figure 4f), the stimulation of cell invasion in response to the VEGF chemoattractant gradient, was inhibited by POE at 8 µg/mL.

To better understand the mechanisms by which POE counteracts the VEGF-mediated pro-invasive and pro-angiogenic effects, we performed Western blot analysis to investigate key signaling pathways involved in endothelial cell invasiveness and angiogenesis. Specifically, we analyzed the phosphorylation status of AKT, ERK, mTOR, and the VEGF receptor 2 (KDR/VEGFR2).

We observed that total KDR levels are elevated in cells treated with POE alone and with POE + VEGF, compared to control and VEGF-only conditions. In contrast, phosphorylated KDR (p-KDR) levels increase with VEGF alone and also appear elevated in the POE + VEGF condition when viewed without normalization. However, when p-KDR levels are normalized to both total KDR and GAPDH, we find that p-KDR is significantly increased only with VEGF treatment, and not in the POE + VEGF group, where it is notably reduced (Figure 4g,h). This suggests that POE leads to an accumulation of the total (not-phosphorylated) KDR protein, possibly by stabilizing the receptor or reducing its degradation, regardless of VEGF stimulation. At the same time, POE appears to inhibit the phosphorylation (activation) of KDR, even in the presence of VEGF. Therefore, although the absolute amount of p-KDR may look similar between VEGF and POE + VEGF conditions, the relative activation of the receptor (i.e., the fraction of phosphorylated KDR relative to the total receptor) is markedly reduced when POE is present. This indicates that POE interferes with KDR activation rather than its expression, ultimately leading to a functional suppression of VEGF signaling despite the presence of increased receptor levels, contributing to the observed inhibition of angiogenic and invasive responses. This supports the idea that POE inhibits VEGF-induced receptor activation, not by downregulating KDR expression, but by preventing its activation, thereby blocking downstream pro-angiogenic signaling.

Similarly, the phosphorylation of mTOR (p-mTOR), a key regulator of cell growth, metabolism, and angiogenesis, was significantly upregulated by VEGF stimulation. However, co-treatment with POE resulted in a noticeable reduction in p-mTOR expression, reinforcing the notion that POE suppresses VEGF-driven pro-angiogenic signaling at multiple levels.

In contrast, AKT phosphorylation (p-AKT) was increased by VEGF and remained elevated even in the presence of POE. This indicates that POE does not inhibit AKT activation and suggests a selective modulation of VEGF signaling rather than a broad suppression. The persistent activation of AKT, despite the anti-angiogenic effects of POE, may reflect a compensatory or survival mechanism. Interestingly, although p-AKT levels remain elevated in the presence of both POE and VEGF, the phosphorylation of mTOR (p-mTOR), which lies downstream of AKT in the signaling cascade, is significantly reduced under the same conditions. This dissociation between AKT and mTOR activation suggests that POE interferes with the functional output of the AKT pathway, selectively blocking downstream signaling despite sustained AKT phosphorylation.

As for ERK phosphorylation (p-ERK), it was also enhanced by VEGF and resulted-reduced when POE was added.

Taken together, these data suggest that POE selectively disrupts VEGF signaling in ECFCs, particularly through the inhibition of p-KDR, p-mTOR and p-ERK, while leaving other pathways like p-AKT relatively unaffected. This selective modulation indicates that POE does not act as a general kinase inhibitor, but rather exerts a targeted modulation of VEGF signaling, particularly by dampening the pro-angiogenic arm of the pathway, despite the continued presence of VEGF.

### 3.5. Effect of POE on Endothelial Activation

Given the established relationship between inflammation and oxidative stress, where ROS production is both a consequence and a driver of inflammatory responses, we next assessed the potential anti-inflammatory effects of POE [28,29]. Our results showed a significant reduction in VEGF induced ROS production following POE treatment, which correlated with a marked decrease in inflammatory markers.

To assess whether treatment with POE induces endothelial activation in ECFCs, the expression of genes involved in inflammatory and coagulation pathways was evaluated by quantitative Real-Time PCR. Tumor Necrosis Factor alpha (TNF-α), a known inducer of both inflammatory and pro-coagulant responses, was used as a positive control.

The genes analyzed included *VCAM-1*, *ICAM-1*, and *Tissue Factor (TF)*. VCAM-1 and ICAM-1 are pro-inflammatory adhesion molecules: VCAM-1 facilitates the adhesion of monocytes, eosinophils, and basophils to the endothelial surface, while ICAM-1 plays a crucial role in the adhesion of macrophages and lymphocytes to endothelial cells. Under physiological conditions, both genes are upregulated in response to cytokines such as TNF-α, which triggers inflammatory endothelial activation.

Pro-coagulant activation was assessed through the expression of Tissue Factor, a key initiator of the coagulation cascade.

**Figure 5:**
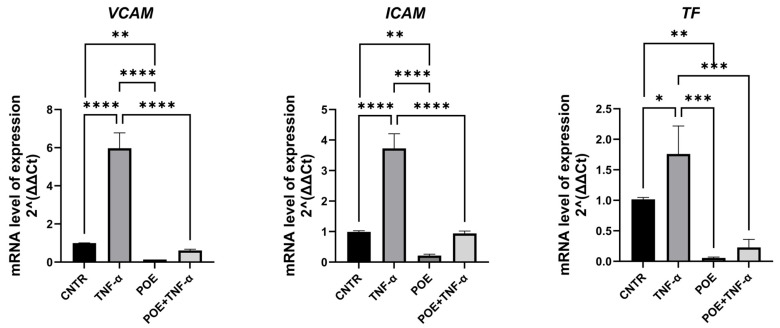
ECFC expression of endothelial activation gene after POE treatment. Relative mRNA expression levels of *VCAM*, *ICAM* and *TF* were analyzed by quantitative real-time PCR in ECFCs under different treatment conditions: control, POE alone (8 μg/mL), TNF-α (10 ng/mL) and TNF-α combined with POE. Gene expression levels were normalized to 18S and expressed as fold change relative to control. Data are presented as mean ± SD of at least three independent experiments. Statistical significance was assessed by one-way ANOVA. * *p* < 0.05; ** *p* < 0.01; *** *p* < 0.001; **** *p* < 0.0001.

As shown in Figure 5, the expression levels of these genes were lower in ECFCs treated with POE at 8 µg/mL, compared to the relative control. Furthermore, in cells co-treated with TNF-α and POE, the extract was able to partially reverse the TNF-α-induced upregulation of these endothelial activation markers, indicating that POE may counteract both inflammatory and pro-coagulant endothelial activation.

## 4. Discussion

Angiogenesis is a critical physiological process not only for embryonic development and reproductive function, but also for tissue repair and regeneration [1]. Nevertheless, its dysregulation is implicated in the pathophysiology of numerous diseases, including cancer, ocular disorders, psoriasis, and chronic inflammatory conditions [2,3]. The dual nature of angiogenesis, being both essential and potentially harmful, has led to a growing interest in understanding its regulatory mechanisms and therapeutic modulation.

Endothelial cells and their progenitors, particularly Endothelial Colony-Forming Cells, have emerged as important players in vascular biology. Their accessibility from sources such as umbilical cord blood and their proliferative potential enhance their translational relevance and renders them valuable tools for angiogenesis studies [30].

Targeting the angiogenic process, particularly through the inhibition of endothelial cell function, has proven to be a viable strategy for restricting tumor growth by limiting the supply of oxygen and nutrients and for the treatment of diseases characterized by an aberrant angiogenesis. Currently, the anti-angiogenic pharmacological arsenal is dominated by monoclonal antibodies, particularly those directed against VEGF or its receptors [6,7]. While effective in some clinical settings, these agents are often associated with significant drawbacks, including high costs and adverse effects such as hemorrhage, most notably observed with Bevacizumab. Moreover, although VEGFR2 tyrosine kinase inhibitors appear to carry a lower bleeding risk, the precise mechanisms underlying their toxicity profiles remain poorly understood [9].

Given the limitations of conventional anti-angiogenic therapies, interest has shifted toward naturally derived compounds capable of modulating angiogenesis through multitargeted mechanisms [13]. Natural products offer complex chemical scaffolds that may exert synergistic effects on angiogenic pathways, including the inhibition of endothelial cell proliferation and migration, induction of apoptosis, and interference with tumor-immune system interactions [14]. Although preclinical studies have provided promising results in both in vitro and in vivo models, the transition to clinical application remains a significant challenge. Further research is required to determine whether individual compounds or complex phytochemical mixtures will offer the best therapeutic potential.

Our results contribute to the growing body of evidence supporting the anti-invasive and anti-angiogenic potential of the marine plant *Posidonia oceanica* [16]. In recent years, this species has attracted increasing attention in biomedical research due to its rich content of marine-derived bioactive compounds, particularly polyphenols, which confer a range of biological activities potentially beneficial for human health. 

In this study, we isolated the hydrophilic fraction from a hydroethanolic extract of *P. oceanica* leaves, yielding a bioactive extract optimized for in vitro experimentation according to a well-established and validated protocol [22].

As a first step, we examined the morphology of ECFCs treated with POE using standard light microscopy followed by confocal microscopy (Figure 1). Cells were stained with phalloidin, a fluorescent marker for cytoskeletal actin filaments, and no significant morphological differences were observed between POE-treated cells and untreated controls.

We then assessed the in vitro effects of POE on ECFC viability (Figure 1). The viability of treated cells was comparable to that of untreated control cells, suggesting that the extract does not exert cytotoxic effects on ECFCs. Interestingly, higher concentrations of the extract appeared to promote a slight, albeit not statistically significant, increase in cellular well-being and proliferation. This observation may be attributed to the antioxidant properties of the polyphenols present in POE, which may help protect cells from oxidative stress.

Cell migration and invasiveness are essential features not only in physiological processes such as angiogenesis and wound healing, but also in pathological conditions, especially in the metastatic cascade of tumor cells. These processes allow cells to degrade components of the extracellular matrix, invade surrounding tissue, and migrate from the primary tumor site. Importantly, endothelial cell invasiveness is also a key step during both physiological and tumor-associated angiogenesis [1].

Based on prior findings showing that *P. oceanica* inhibits the invasive and migratory behavior of cancer cells [21], we evaluated whether the extract could exert similar effects on ECFCs [31]. To assess this, we performed a Transwell invasion and scratch assays (Figure 2). The results clearly demonstrated that POE significantly reduced the migration and invasive ability of ECFCs. Specifically, we observed a marked decrease in the number of cells able to invade a Geltrex-coated membrane, which mimics the extracellular matrix in vitro. Furthermore, using a scratch wound healing assay, we found that POE significantly impaired ECFC migration, further confirming its impact on key steps of angiogenesis.

In addition to its anti-invasive/migration effects, POE also inhibited the ability of ECFCs to form capillary-like tubular structures in a dose-dependent manner, as assessed by a tube formation assay (Figure 2). This finding strongly supports the anti-angiogenic potential of the extract.

Our findings reveal that POE effectively interferes with VEGF-induced angiogenic processes in human ECFCs, expanding on its previously described anti-migratory and antioxidant properties [32]. Unaltered viability and morphology at the highest dose (8 µg/mL) support POE’s safety profile, aligning with reports of its nontoxic antioxidant and anti-inflammatory actions in various cell models. The dose-dependent suppression of ECFC migration, invasion, and tube formation extends earlier observations of POE inhibiting cancer cell motility via downregulation of MMP-2/9 and induction of autophagy.

Mechanistically, we show for the first time that POE disrupts VEGF signaling in ECFCs by stabilizing total KDR without promoting its phosphorylation, thus decreasing the p-KDR-to-KDR ratio (Figure 4). This phenomenon suggests receptor inactivation without downregulation of receptor levels, a novel mechanism for POE. Concurrently, VEGF-induced phosphorylation of mTOR and p-ERK was significantly attenuated, despite sustained p-AKT levels, indicating a selective blockade downstream of AKT that spares general survival signaling. The suppression of ERK activation, a key mediator of endothelial proliferation and migration, further highlights POE’s targeted interference with pro-angiogenic signaling. This differential targeting suggests that POE does not act as a broad kinase inhibitor but rather as a precise modulator of pro-angiogenic signaling.

VEGF functions also as a potent chemoattractant that orchestrates the directed migration of endothelial cells (ECs) through the extracellular matrix (ECM), a critical step in the angiogenic cascade. This chemotactic response, mediated by ECs sensing and migrating along a VEGF concentration gradient, plays a pivotal role in sprouting angiogenesis and the formation of functional neovessels [1]. Here, we showed that POE is able to counteract even the chemoinvasion VEGF mediated (Figure 4).

In light of the findings described above demonstrating a dose-dependent inhibition of ECFC angiogenic functions, we selected 8 µg/mL POE as the most effective concentration for subsequent experiments. We showed that POE effectively mitigated the pro-oxidant effects elicited by VEGF stimulation, suggesting a potential antioxidant role of POE in modulating redox homeostasis in ECFCs (Figure 3).

Our findings indicate that POE not only interferes with key functional properties of ECFCs, such as migration, invasion, and vascular structure formation, but also modulates redox-related gene expression in response to VEGF-induced oxidative stress. In particular, the antioxidant and detoxifying genes *hTRX1*, *hTRX2*, *PRDX2*, *AKR1C1*, and *AKR1B10* were downregulated following POE treatment, while they were significantly upregulated upon VEGF stimulation (Figure 3). This pattern is consistent with a cellular response to increased ROS levels driven by pro-angiogenic stimuli. Thioredoxins (*hTRX1* and *hTRX2*) and *PRDX2* are central components of the cellular redox buffering system, essential for endothelial survival and function under oxidative conditions. Similarly, *AKR1C1* and *AKR1B10* contribute to the detoxification of lipid peroxidation-derived aldehydes, which are elevated during oxidative stress and can interfere with endothelial integrity. The observed antioxidant effect, evidenced by reduced ROS and downregulation of redox-sensitive genes, is consistent with POE’s high phenolic content, including chicoric and chlorogenic acids. Supporting data include inhibition of ROS and NO in LPS-stimulated macrophages via ERK/AKT/NF-κB suppression [32].

The observation that POE co-treatment attenuates the VEGF-induced overexpression of these genes suggests that the extract may act upstream by reducing intracellular ROS accumulation. This is in line with our ROS quantification data and points to a direct antioxidant action of POE, potentially limiting the activation of ROS-sensitive signaling pathways such as those governed by Nrf2 or MAPKs. The ability of POE to restore redox balance under pro-angiogenic conditions highlights its potential as a natural modulator of endothelial oxidative stress and reinforces its candidacy as an anti-angiogenic agent. Further studies will be needed to delineate the precise molecular mechanisms involved and to identify the bioactive constituents responsible for these effects.

In the past two decades, angiogenesis-targeted therapies have emerged as a major class of treatments for cancer and other diseases characterized by aberrant neovascularization. The vascular endothelial growth factor (VEGF) family and its receptors represent key molecular targets within this therapeutic strategy. To 2021, 16 anti-angiogenic agents have been approved in the United States for cancer treatment, with many more currently under development [33].

However, anti-angiogenic therapies are associated with significant concerns regarding toxicity and adverse effects, particularly the increased risk of thrombotic and hemorrhagic events. These complications have become major clinical concerns, especially in oncology patients or individuals with pre-existing risks for thrombosis and bleeding [34].

To investigate whether POE may elicit endothelial activation, we analyzed the expression of genes related to inflammatory and coagulation pathways in ECFCs using quantitative Real-Time PCR. Specifically, we assessed the expression of VCAM-1 and ICAM-1, two key pro-inflammatory adhesion molecules, and Tissue Factor, a primary initiator of the coagulation cascade (Figure 5).

Our findings indicate that in ECFCs co-treated with the pro-inflammatory and pro-coagulant cytokine TNF-α and POE, the extract effectively counteracted the TNF-α-induced upregulation of target genes. These promising results pave the way for further studies aimed at evaluating the safety and therapeutic potential of POE as an anti-angiogenic agent, with particular attention to its effects on inflammation and coagulation pathways.

A schematic representation of the proposed mechanism of action of POE on ECFCs, integrating its antioxidant, anti-inflammatory, and anti-angiogenic effects, is provided in the Graphical Abstract. This diagram summarizes the multitarget modulation exerted by POE on VEGF signaling, redox balance, and endothelial activation, supporting its potential as a natural therapeutic agent against pathological angiogenesis. The observed impairment in ECFC migration, invasiveness, and tube formation highlights the potential of *P. oceanica* as a modulator of angiogenesis. These findings position *P. oceanica* not only as a source of natural anti-angiogenic agents but also as a candidate for combinatorial approaches aimed at overcoming resistance to conventional VEGF-targeted therapies. Taken together, our findings propose *P. oceanica* extract as a promising, low-cost and low-toxicity candidate for further preclinical investigation as adjunctive or alternative therapeutic strategies targeting angiogenesis in both oncological and non-oncological settings. In particular, POE may represent a promising emerging treatment for diseases such as Diabetic Retinopathy (DR), which is characterized by abnormal neovascularization in the retina and is among the leading causes of blindness worldwide. VEGF has a crucial role in the pathogenesis of DR and current therapeutic approaches often present significant limitations and complications.

However, further studies are warranted to identify the specific bioactive constituents responsible for these effects and to assess their efficacy and safety in vivo. The complexity of the extract and the presence of multiple phytochemicals raise the possibility of synergistic interactions, which could be exploited in future formulations. In addition, the standardization of extraction methods and the exploration of structure-activity relationships will be critical for advancing *P. oceanica*-based therapeutics toward clinical translation.

## Figures and Tables

**Table 1:** Polyphenolic composition of *P. oceanica* leaf extract (POE).

Compound	Structure	Percentage (%)
Gallic acid	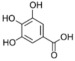	0.374
Chlorogenic acid	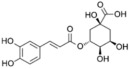	0.639
(+)-Catechin	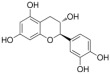	84.762
(−)-Epicatechin	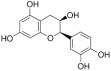	1.383
Ferulic acid	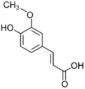	1.729
Undefined	–	11.113

**Table 2:** List of primers.

Gene	Forward	Reverse
*18S*	5′-CCAGTAAGTGCGGGTCATAAG-3′	5′-GCCTCACATAACCATCCAATC-3′
*hTRX1*	5′-CTGCTTTTCAGGAAGCCTTG-3′	5′-TGTTGGCATGCATTTGACTT-3′
*hTRX2*	5′-AGCCCGGACAATATACACCA -3′	5′-AATATCCACCTTGGCCATCA-3′
*PRDX2*	5′-GTCCTTCGCCAGATCACTGTT-3′	5′-CATGCTGGTCTGTGTACTGGA-3′
*AKR1C1*	5′-TGCATAATGCCTGGGCTATCTT-3′	5′-AGGCCATGACAGTGTTTGAG-3′
*AKR1B10*	5′-CCAAGTCTGTGACACCAGCA-3′	5′-CGTTACAGGCCCTCCAGTTT-3′
*VCAM*	5′-GGCGCCTATACCATCCGAAA-3′	5′-GAGCACGAGAAGCTCAGGAGAA-3′
*ICAM*	5′-CTACCTCTGTCGGGCCAGGA-3′	5′-AGGCCTGCAGTGCCCATTA-3′
*TF*	5′-CCCGAACAGTTAACCGGAAGA-3′	5′-GGAGTTCTCCTTCCAGCTCTGC-3′

## Data Availability

The original contributions presented in this study are included in the article/Supplementary Material. Further inquiries can be directed to the corresponding author(s).

## Supplementary Materials

The following supporting information can be downloaded at: https://www.mdpi.com/article/10.3390/jox15050153/s1, Figure S1: Original Western blot images of p-KDR, total KDR and GAPDH reported in Figure 4g; Figure S2: original Western blot images of p-AKT, total AKT and GAPDH reported in Figure 4g; Figure S3: original Western blot images of p-mTOR, mTOR, p-ERK, total ERK and GAPDH reported Figure 4g; File S1: Original images of Figure 1a,b, Figure 2a,c,e, Figure 4a,c,e.

## Abbreviations

The following abbreviations are used in this manuscript:

AKR1B10	Aldo-Keto Reductase Family 1 Member B10
AKR1C1	Aldo-Keto Reductase Family 1 Member C1
AKT	Protein Kinase B
AMD	Age-Related Macular Degeneration
DAPI	4′,6-Diamidino-2-Phenylindole
DMEM	Dulbecco’s Modified Eagle Medium
DMSO	Dimethyl Sulfoxide
ECFC	Endothelial Colony-Forming Cell
ECM	Extracellular Matrix
EGM-2	Endothelial Growth Medium-2
ERK	Extracellular Signal-Regulated Kinase
EtOH	Ethanol
FBS	Fetal Bovine Serum
GAPDH	Glyceraldehyde-3-Phosphate Dehydrogenase
H_2_O_2_	Hydrogen Peroxide
hTRX1	Human Thioredoxin 1
hTRX2	Human Thioredoxin 2
ICAM-1	Intercellular Adhesion Molecule 1
KDR	Kinase Insert Domain Receptor (VEGFR2)
LPS	Lipopolysaccharide
MAPKs	Mitogen-Activated Protein Kinases
MMP 2/9	Matrix Metalloproteinases 2 and 9
mTOR	Mechanistic Target of Rapamycin
MTT	3-(4,5-Dimethylthiazol-2-yl)-2,5-Diphenyltetrazolium Bromide
NF-κB	Nuclear Factor kappa-light-chain-enhancer of activated B cells
NO	Nitric Oxide
PBS	Phosphate-Buffered Saline
p-AKT	Phosphorylated AKT
PCR	Polymerase Chain Reaction
p-ERK	Phosphorylated ERK
p-KDR	Phosphorylated KDR
POE	Posidonia oceanica Extract
PRDX2	Peroxiredoxin 2
p-MTOR	Phosphorylated mTOR
ROS	Reactive Oxygen Species
TF	Tissue Factor
TNF-α	Tumor Necrosis Factor Alpha
TP	Total Polyphenol
UCB	Umbilical Cord Blood
ULEX	Ulex Europaeus Agglutinin
VCAM-1	Vascular Cell Adhesion Molecule 1
VEGF	Vascular Endothelial Growth Factor
VEGFR	Vascular Endothelial Growth Factor Receptor
vWF	von Willebrand Factor
