# Supplementary material for: Posidonia oceanica Extract Inhibits VEGF-Induced Angiogenic and Oxidative Responses in Human Endothelial Colony-Forming Cells
Source: J Xenobiot. 2025 Sep 17;15(5):153. doi: 10.3390/jox15050153 (PMC12452316; doi:10.3390/jox15050153)
Supplement: Supplementary file 1 [file jox-15-00153-s001.zip › jox-3780983-Supplementary materials-done-send XML.pdf]

Journal of Xenobiotics

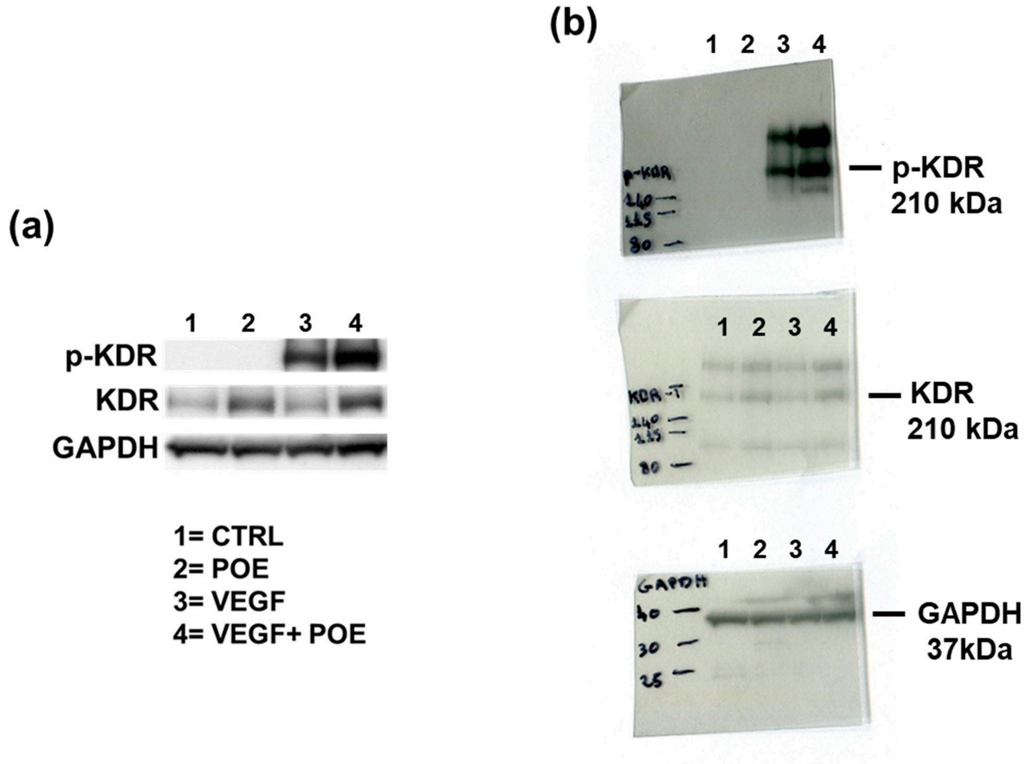

Figure S1. **(a)** Western blot images included in the manuscript (Figure 4g). **(b)** Related original Western blot images of p-KDR, total KDR and GAPDH reported as loading control. ECFCs were treated with **2**- POE (8 μg/mL), **3**- VEGF (50 ng/ml) and **4**- with the combination of VEGF and POE.

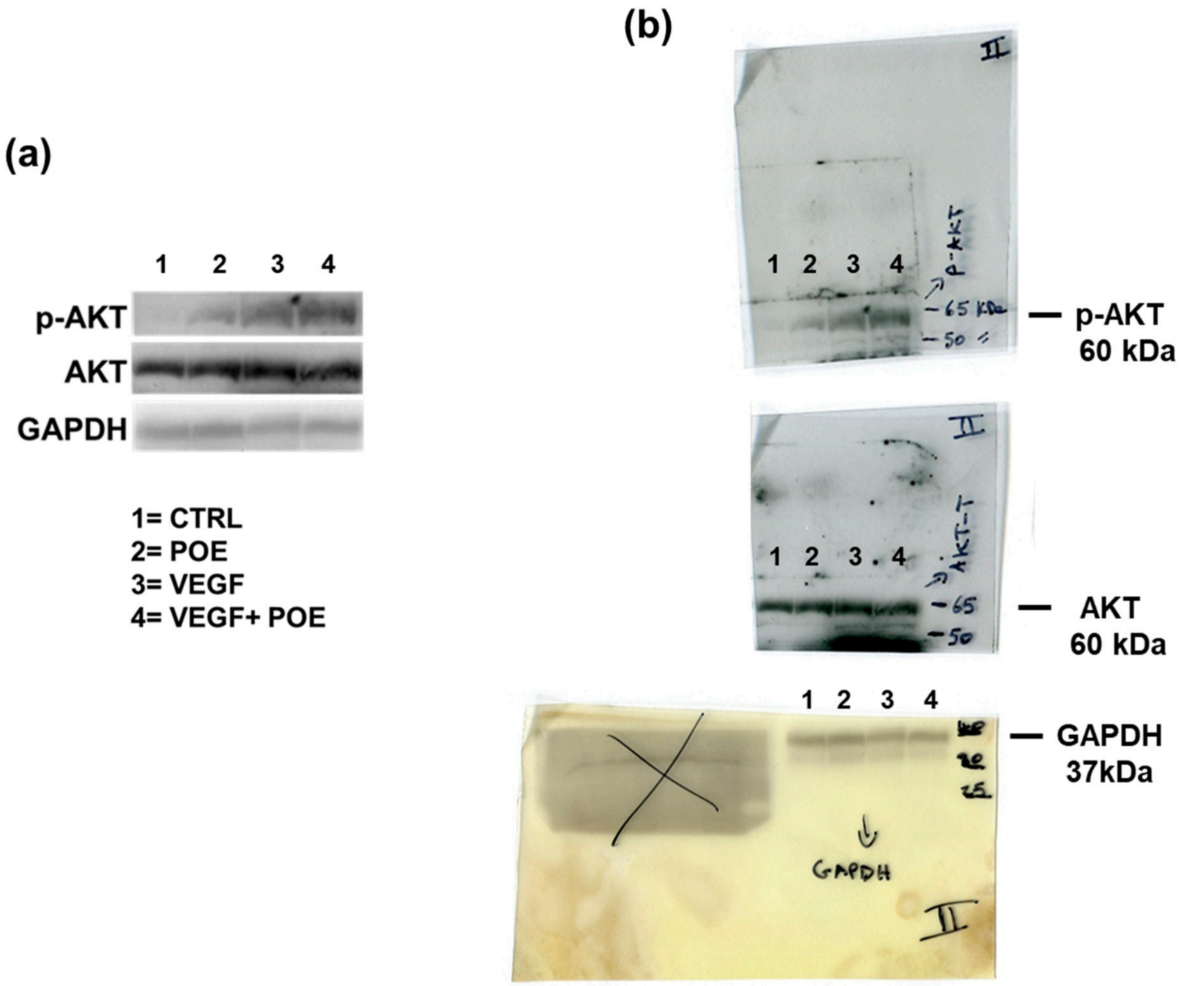

Figure S2. (a) Western blot images included in the manuscript (Figure 4g). **(b)** Related original Western blot images of p-AKT, total AKT and GAPDH reported as loading control. ECFCs were treated with **2**- POE (8 µg/mL), **3**- VEGF (50 ng/ml) and **4**- with the combination of VEGF and POE.

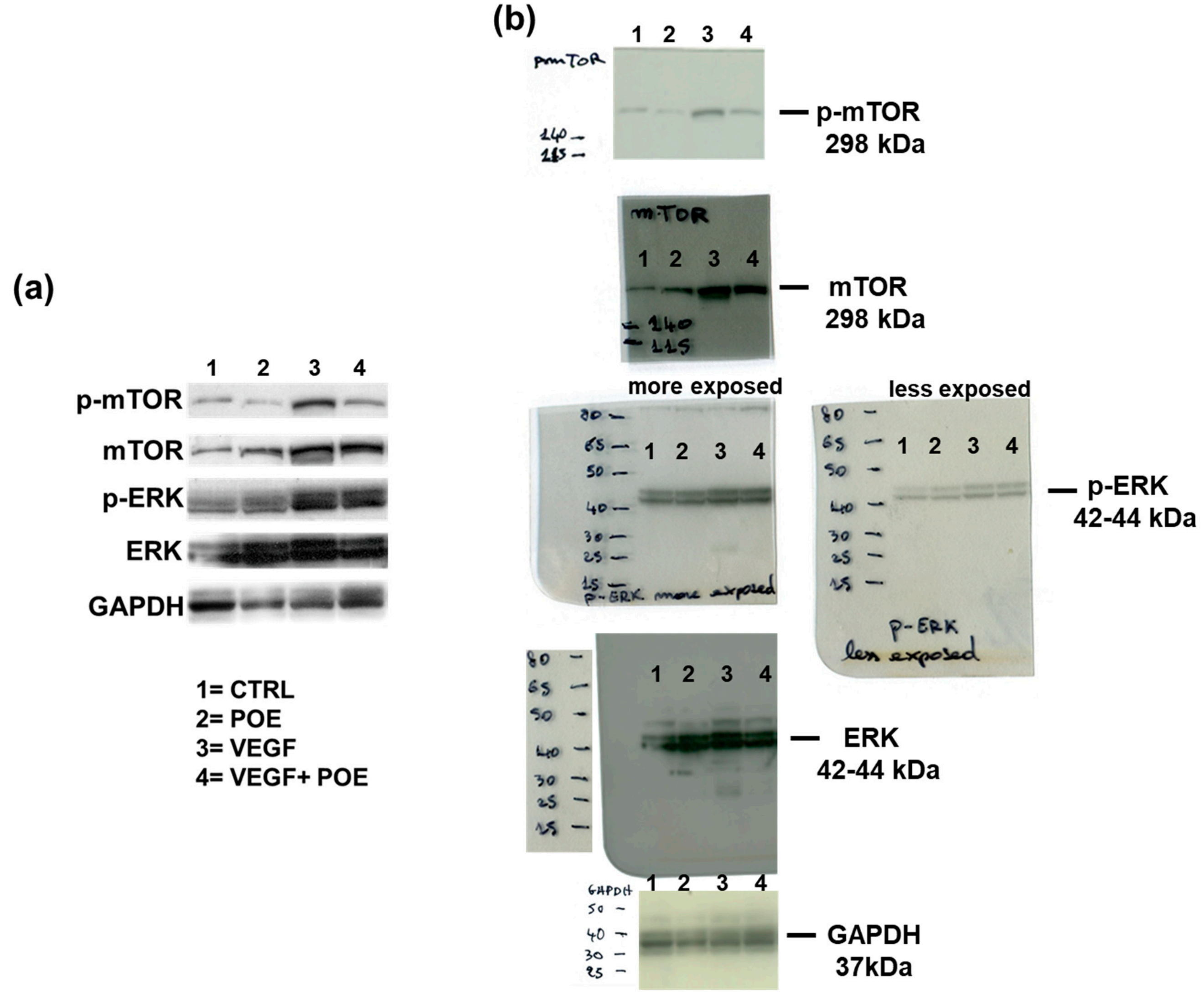

Figure S3. (a) Western blot images included in the manuscript (Figure 4g). **(b)** Related original Western blot images of p-mTOR, mTOR, p-ERK (more and less exposed), total ERK and GAPDH reported as loading control. ECFCs were treated with **2**- POE (8 µg/mL), **3**- VEGF (50 ng/ml) and **4**- with the combination of VEGF and POE.
