# Supplementary figures and images for: Posidonia oceanica Extract Inhibits VEGF-Induced Angiogenic and Oxidative Responses in Human Endothelial Colony-Forming Cells
Source: J Xenobiot. 2025 Sep 17;15(5):153. doi: 10.3390/jox15050153 (PMC12452316; doi:10.3390/jox15050153)

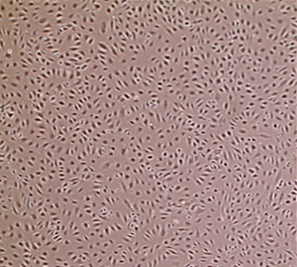

Supplement: Supplementary file 1 [file jox-15-00153-s001.zip › File S1-Original images of Figures 1a, 1b, 2a, 2c, 2e, 4a, 4c and 4e/Figure 1a/CTRL.tif]

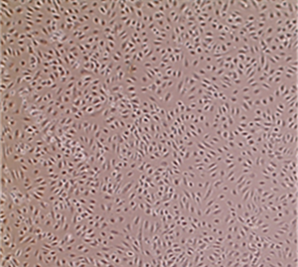

Supplement: Supplementary file 1 [file jox-15-00153-s001.zip › File S1-Original images of Figures 1a, 1b, 2a, 2c, 2e, 4a, 4c and 4e/Figure 1a/POE 4.tif]

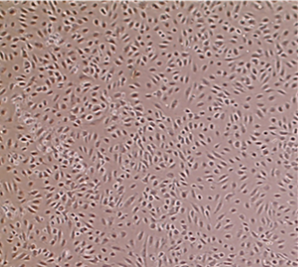

Supplement: Supplementary file 1 [file jox-15-00153-s001.zip › File S1-Original images of Figures 1a, 1b, 2a, 2c, 2e, 4a, 4c and 4e/Figure 1a/POE 6.tif]

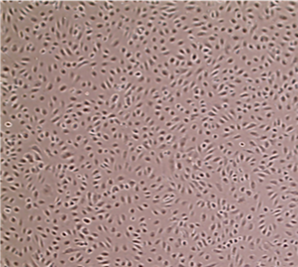

Supplement: Supplementary file 1 [file jox-15-00153-s001.zip › File S1-Original images of Figures 1a, 1b, 2a, 2c, 2e, 4a, 4c and 4e/Figure 1a/POE 8.tif]

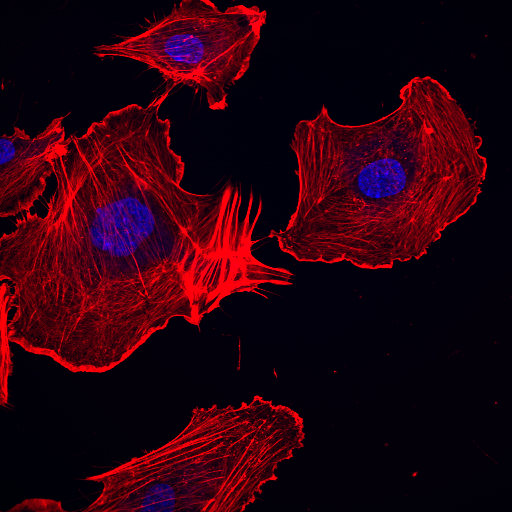

Supplement: Supplementary file 1 [file jox-15-00153-s001.zip › File S1-Original images of Figures 1a, 1b, 2a, 2c, 2e, 4a, 4c and 4e/Figure 1b/CTRL.tif]

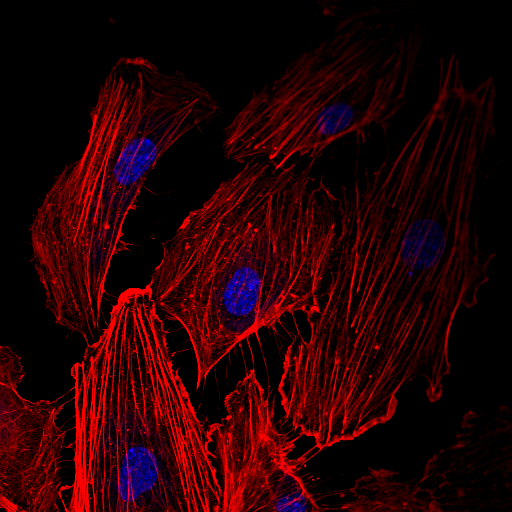

Supplement: Supplementary file 1 [file jox-15-00153-s001.zip › File S1-Original images of Figures 1a, 1b, 2a, 2c, 2e, 4a, 4c and 4e/Figure 1b/POE 4.tif]

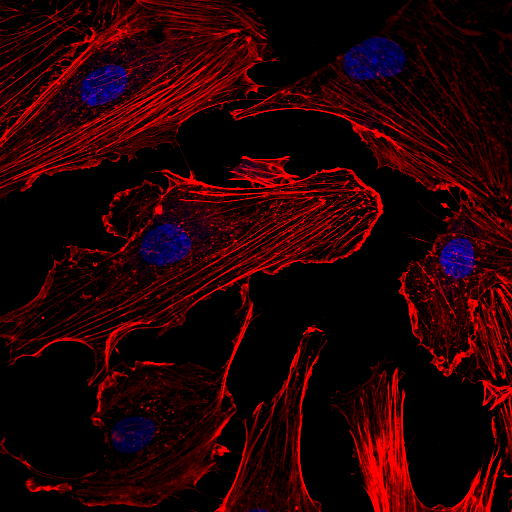

Supplement: Supplementary file 1 [file jox-15-00153-s001.zip › File S1-Original images of Figures 1a, 1b, 2a, 2c, 2e, 4a, 4c and 4e/Figure 1b/POE 6.tif]

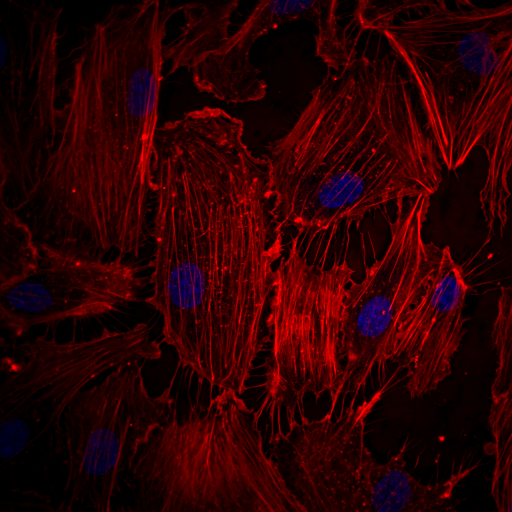

Supplement: Supplementary file 1 [file jox-15-00153-s001.zip › File S1-Original images of Figures 1a, 1b, 2a, 2c, 2e, 4a, 4c and 4e/Figure 1b/POE 8.tif]

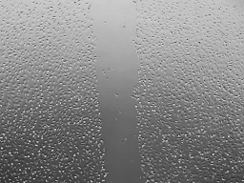

Supplement: Supplementary file 1 [file jox-15-00153-s001.zip › File S1-Original images of Figures 1a, 1b, 2a, 2c, 2e, 4a, 4c and 4e/Figure 2a/CTRL 0h.tif]

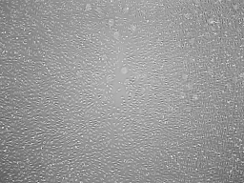

Supplement: Supplementary file 1 [file jox-15-00153-s001.zip › File S1-Original images of Figures 1a, 1b, 2a, 2c, 2e, 4a, 4c and 4e/Figure 2a/CTRL 48h .tif]

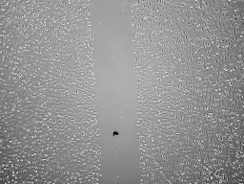

Supplement: Supplementary file 1 [file jox-15-00153-s001.zip › File S1-Original images of Figures 1a, 1b, 2a, 2c, 2e, 4a, 4c and 4e/Figure 2a/POE 4 0h.tif]

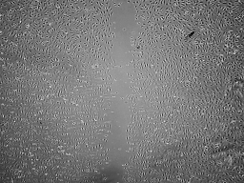

Supplement: Supplementary file 1 [file jox-15-00153-s001.zip › File S1-Original images of Figures 1a, 1b, 2a, 2c, 2e, 4a, 4c and 4e/Figure 2a/POE 4 48h.tif]

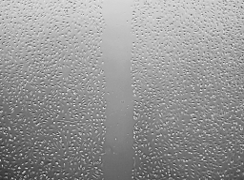

Supplement: Supplementary file 1 [file jox-15-00153-s001.zip › File S1-Original images of Figures 1a, 1b, 2a, 2c, 2e, 4a, 4c and 4e/Figure 2a/POE 6 0h.tif]

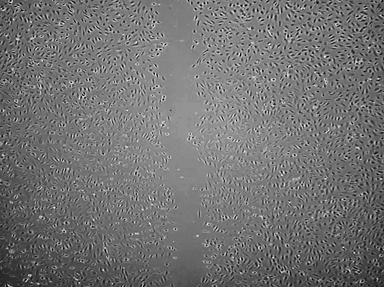

Supplement: Supplementary file 1 [file jox-15-00153-s001.zip › File S1-Original images of Figures 1a, 1b, 2a, 2c, 2e, 4a, 4c and 4e/Figure 2a/POE 6 48h.tif]

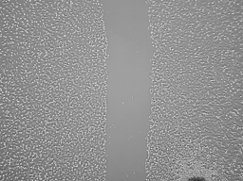

Supplement: Supplementary file 1 [file jox-15-00153-s001.zip › File S1-Original images of Figures 1a, 1b, 2a, 2c, 2e, 4a, 4c and 4e/Figure 2a/POE 8 0h.tif]

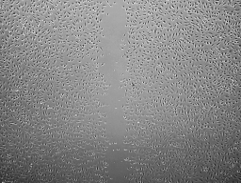

Supplement: Supplementary file 1 [file jox-15-00153-s001.zip › File S1-Original images of Figures 1a, 1b, 2a, 2c, 2e, 4a, 4c and 4e/Figure 2a/POE 8 48h.tif]

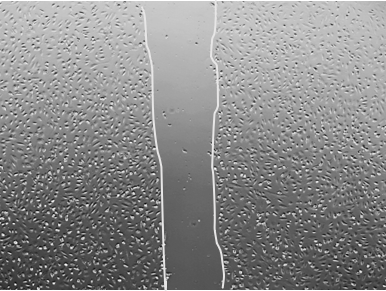

Supplement: Supplementary file 1 [file jox-15-00153-s001.zip › File S1-Original images of Figures 1a, 1b, 2a, 2c, 2e, 4a, 4c and 4e/Figure 2a/with withe line/CTRL 0h.tif]

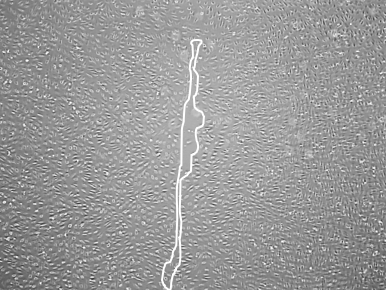

Supplement: Supplementary file 1 [file jox-15-00153-s001.zip › File S1-Original images of Figures 1a, 1b, 2a, 2c, 2e, 4a, 4c and 4e/Figure 2a/with withe line/CTRL 48h.tif]

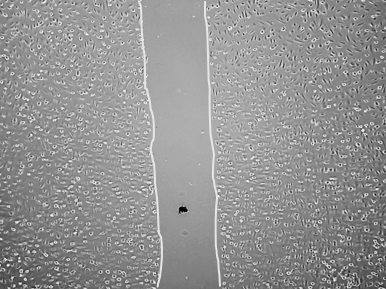

Supplement: Supplementary file 1 [file jox-15-00153-s001.zip › File S1-Original images of Figures 1a, 1b, 2a, 2c, 2e, 4a, 4c and 4e/Figure 2a/with withe line/POE 4 0h.tif]

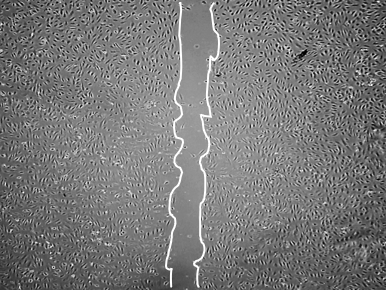

Supplement: Supplementary file 1 [file jox-15-00153-s001.zip › File S1-Original images of Figures 1a, 1b, 2a, 2c, 2e, 4a, 4c and 4e/Figure 2a/with withe line/POE 4 48h.tif]

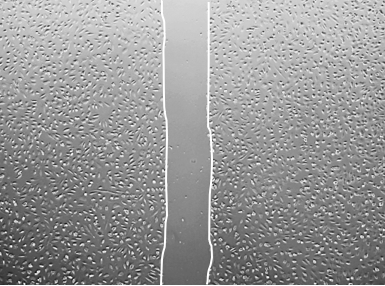

Supplement: Supplementary file 1 [file jox-15-00153-s001.zip › File S1-Original images of Figures 1a, 1b, 2a, 2c, 2e, 4a, 4c and 4e/Figure 2a/with withe line/POE 6 0h.tif]

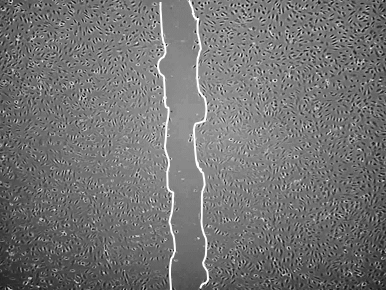

Supplement: Supplementary file 1 [file jox-15-00153-s001.zip › File S1-Original images of Figures 1a, 1b, 2a, 2c, 2e, 4a, 4c and 4e/Figure 2a/with withe line/POE 6 48h.tif]

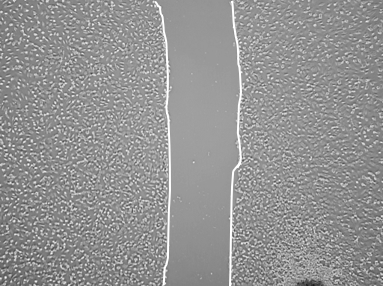

Supplement: Supplementary file 1 [file jox-15-00153-s001.zip › File S1-Original images of Figures 1a, 1b, 2a, 2c, 2e, 4a, 4c and 4e/Figure 2a/with withe line/POE 8 0h.tif]

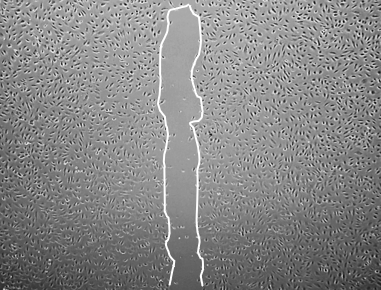

Supplement: Supplementary file 1 [file jox-15-00153-s001.zip › File S1-Original images of Figures 1a, 1b, 2a, 2c, 2e, 4a, 4c and 4e/Figure 2a/with withe line/POE 8 48h.tif]

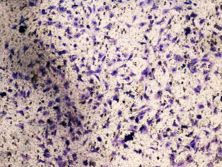

Supplement: Supplementary file 1 [file jox-15-00153-s001.zip › File S1-Original images of Figures 1a, 1b, 2a, 2c, 2e, 4a, 4c and 4e/Figure 2c/CTRL.tif]

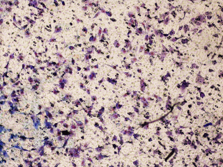

Supplement: Supplementary file 1 [file jox-15-00153-s001.zip › File S1-Original images of Figures 1a, 1b, 2a, 2c, 2e, 4a, 4c and 4e/Figure 2c/POE 4.tif]

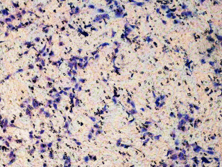

Supplement: Supplementary file 1 [file jox-15-00153-s001.zip › File S1-Original images of Figures 1a, 1b, 2a, 2c, 2e, 4a, 4c and 4e/Figure 2c/POE 6.tif]

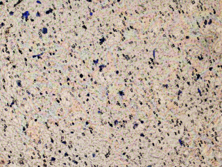

Supplement: Supplementary file 1 [file jox-15-00153-s001.zip › File S1-Original images of Figures 1a, 1b, 2a, 2c, 2e, 4a, 4c and 4e/Figure 2c/POE 8.tif]

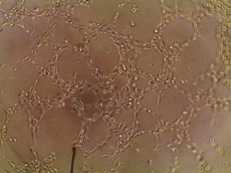

Supplement: Supplementary file 1 [file jox-15-00153-s001.zip › File S1-Original images of Figures 1a, 1b, 2a, 2c, 2e, 4a, 4c and 4e/Figure 2e/CTRL 24h.tif]

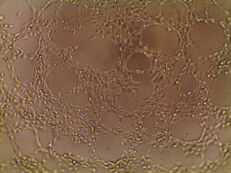

Supplement: Supplementary file 1 [file jox-15-00153-s001.zip › File S1-Original images of Figures 1a, 1b, 2a, 2c, 2e, 4a, 4c and 4e/Figure 2e/CTRL 6h.tif]

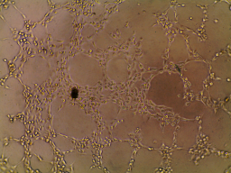

Supplement: Supplementary file 1 [file jox-15-00153-s001.zip › File S1-Original images of Figures 1a, 1b, 2a, 2c, 2e, 4a, 4c and 4e/Figure 2e/POE 4 24h.tif]

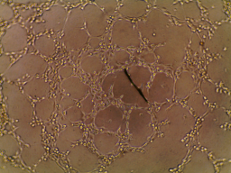

Supplement: Supplementary file 1 [file jox-15-00153-s001.zip › File S1-Original images of Figures 1a, 1b, 2a, 2c, 2e, 4a, 4c and 4e/Figure 2e/POE 4 6h.tif]

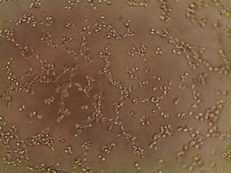

Supplement: Supplementary file 1 [file jox-15-00153-s001.zip › File S1-Original images of Figures 1a, 1b, 2a, 2c, 2e, 4a, 4c and 4e/Figure 2e/POE 6 24h.tif]

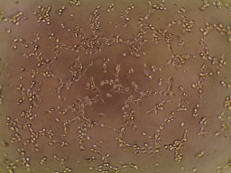

Supplement: Supplementary file 1 [file jox-15-00153-s001.zip › File S1-Original images of Figures 1a, 1b, 2a, 2c, 2e, 4a, 4c and 4e/Figure 2e/POE 6 6h.tif]

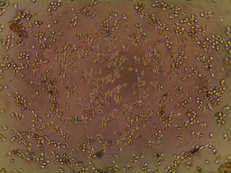

Supplement: Supplementary file 1 [file jox-15-00153-s001.zip › File S1-Original images of Figures 1a, 1b, 2a, 2c, 2e, 4a, 4c and 4e/Figure 2e/POE 8 24h.tif]

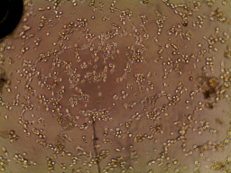

Supplement: Supplementary file 1 [file jox-15-00153-s001.zip › File S1-Original images of Figures 1a, 1b, 2a, 2c, 2e, 4a, 4c and 4e/Figure 2e/POE 8 6h.tif]

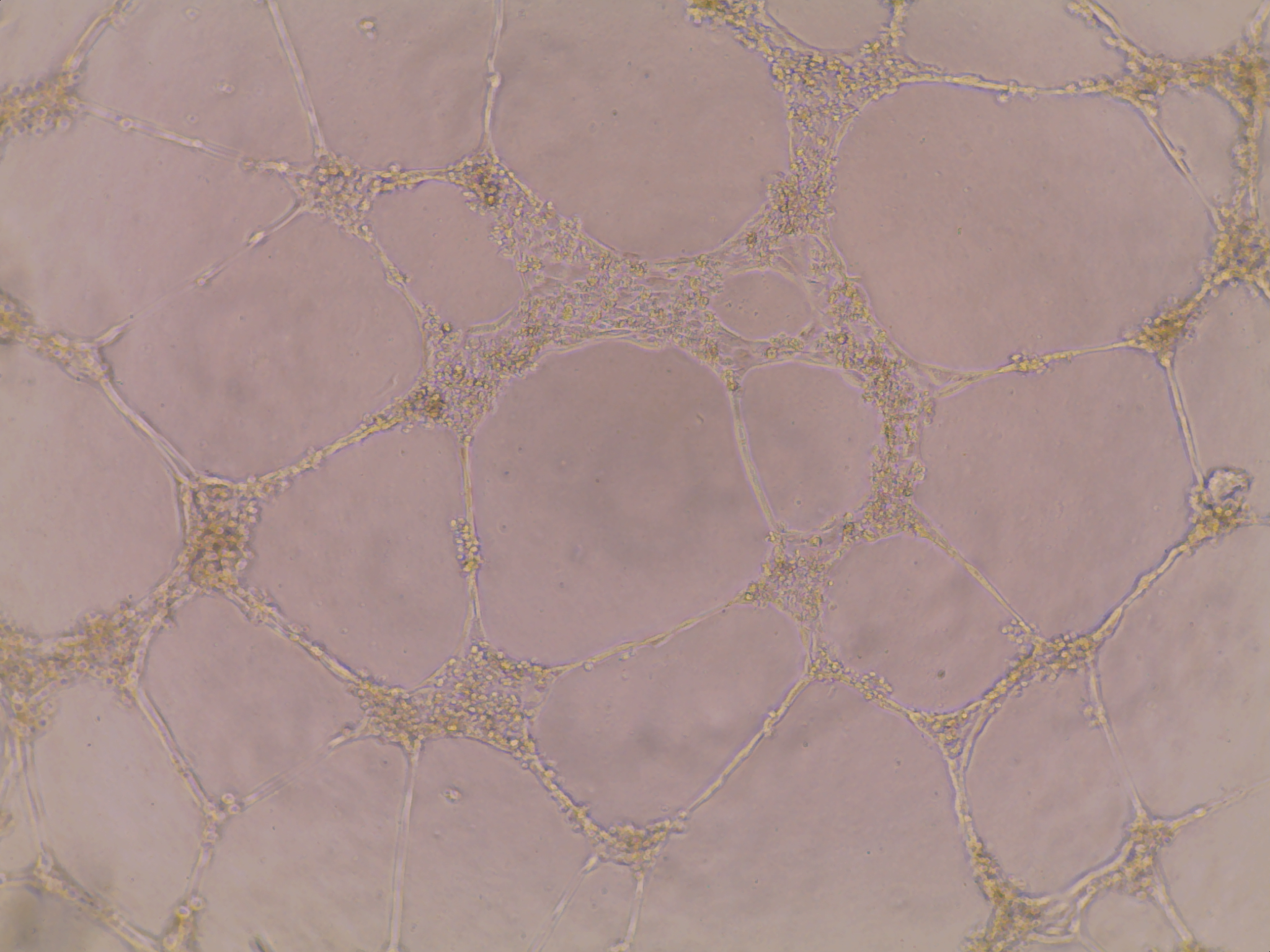

Supplement: Supplementary file 1 [file jox-15-00153-s001.zip › File S1-Original images of Figures 1a, 1b, 2a, 2c, 2e, 4a, 4c and 4e/Figure 4a/CTRL 6h.tif]

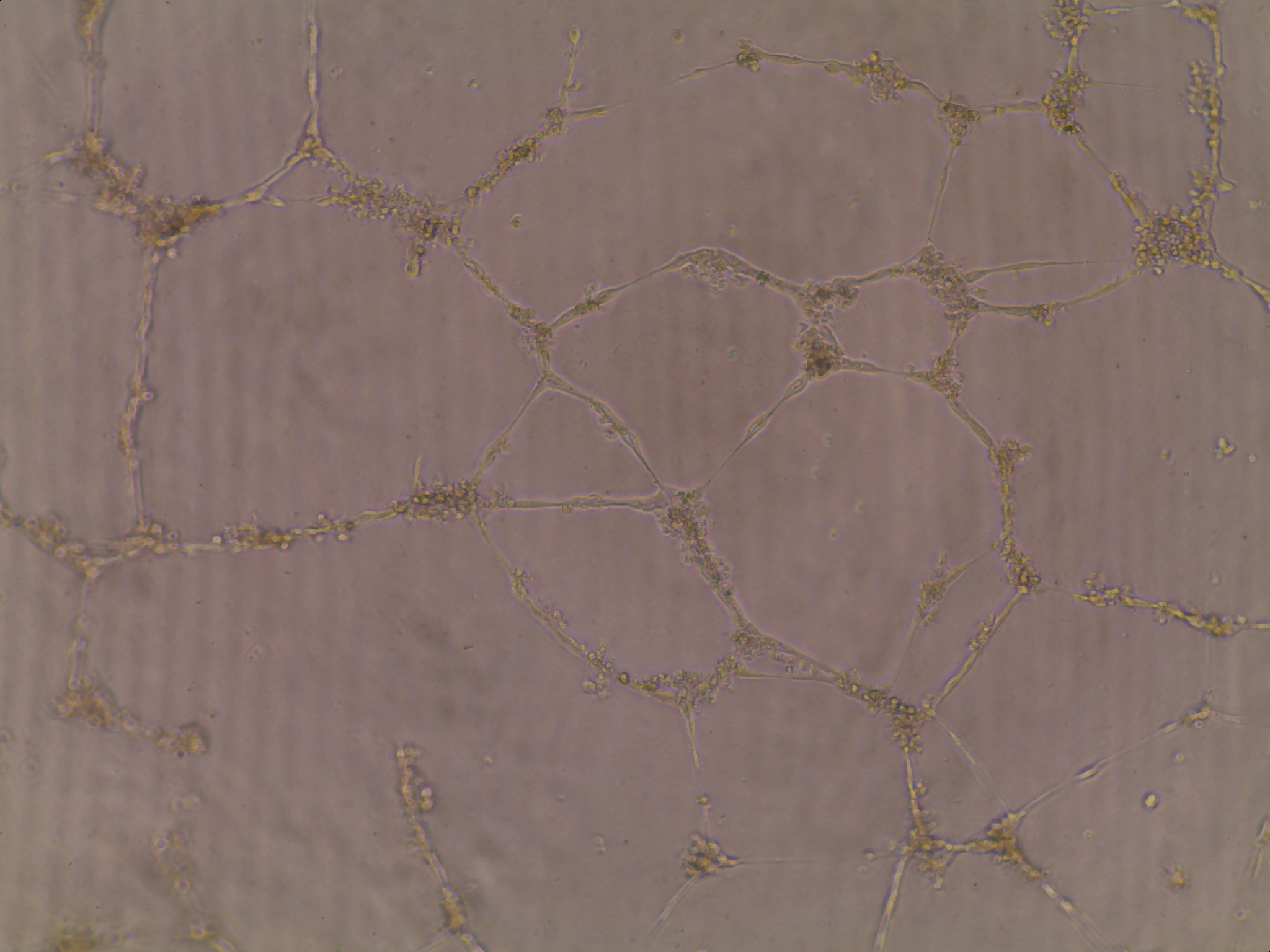

Supplement: Supplementary file 1 [file jox-15-00153-s001.zip › File S1-Original images of Figures 1a, 1b, 2a, 2c, 2e, 4a, 4c and 4e/Figure 4a/POE 6h.tif]

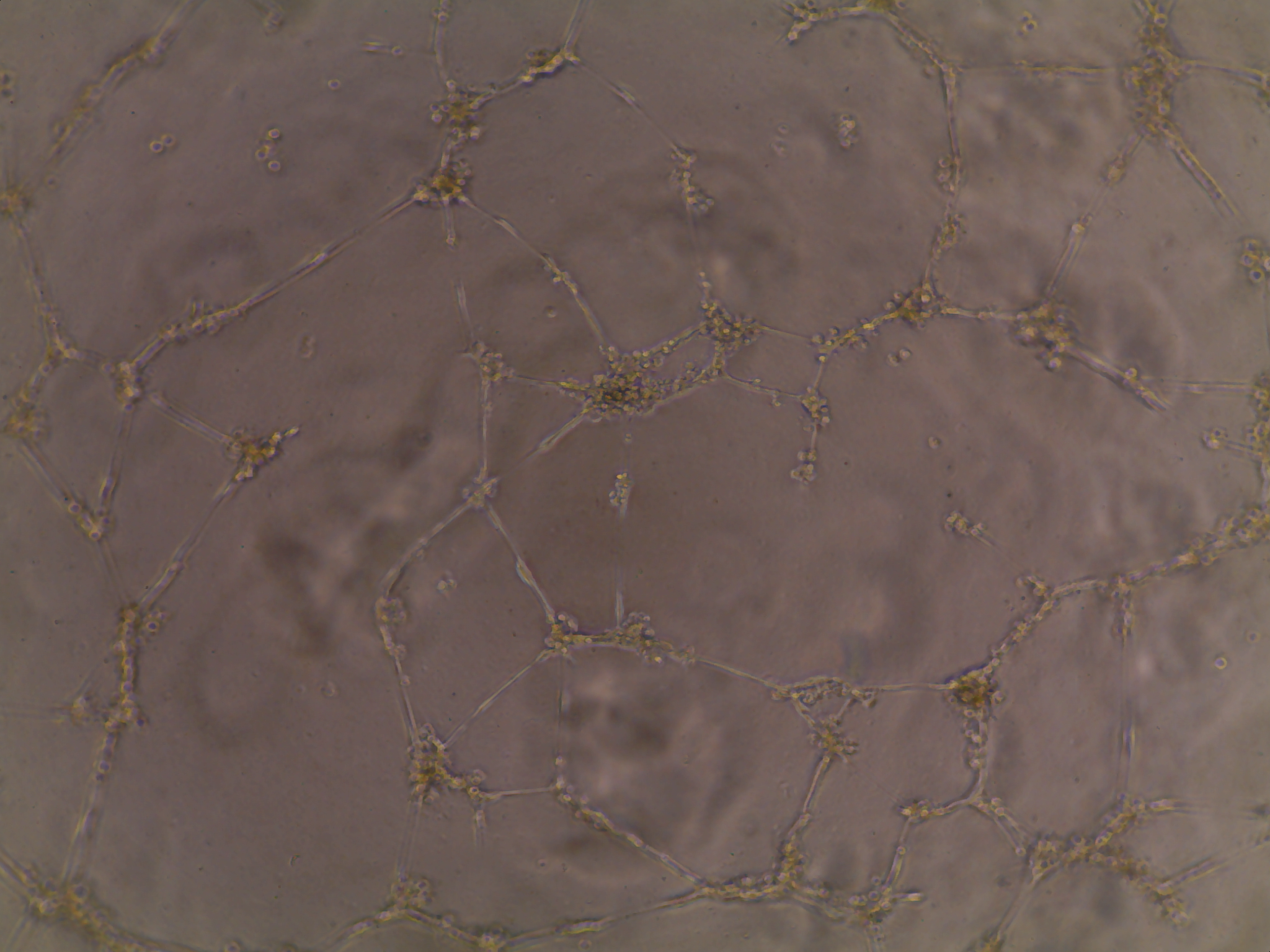

Supplement: Supplementary file 1 [file jox-15-00153-s001.zip › File S1-Original images of Figures 1a, 1b, 2a, 2c, 2e, 4a, 4c and 4e/Figure 4a/POE+VEGF 6h.tif]

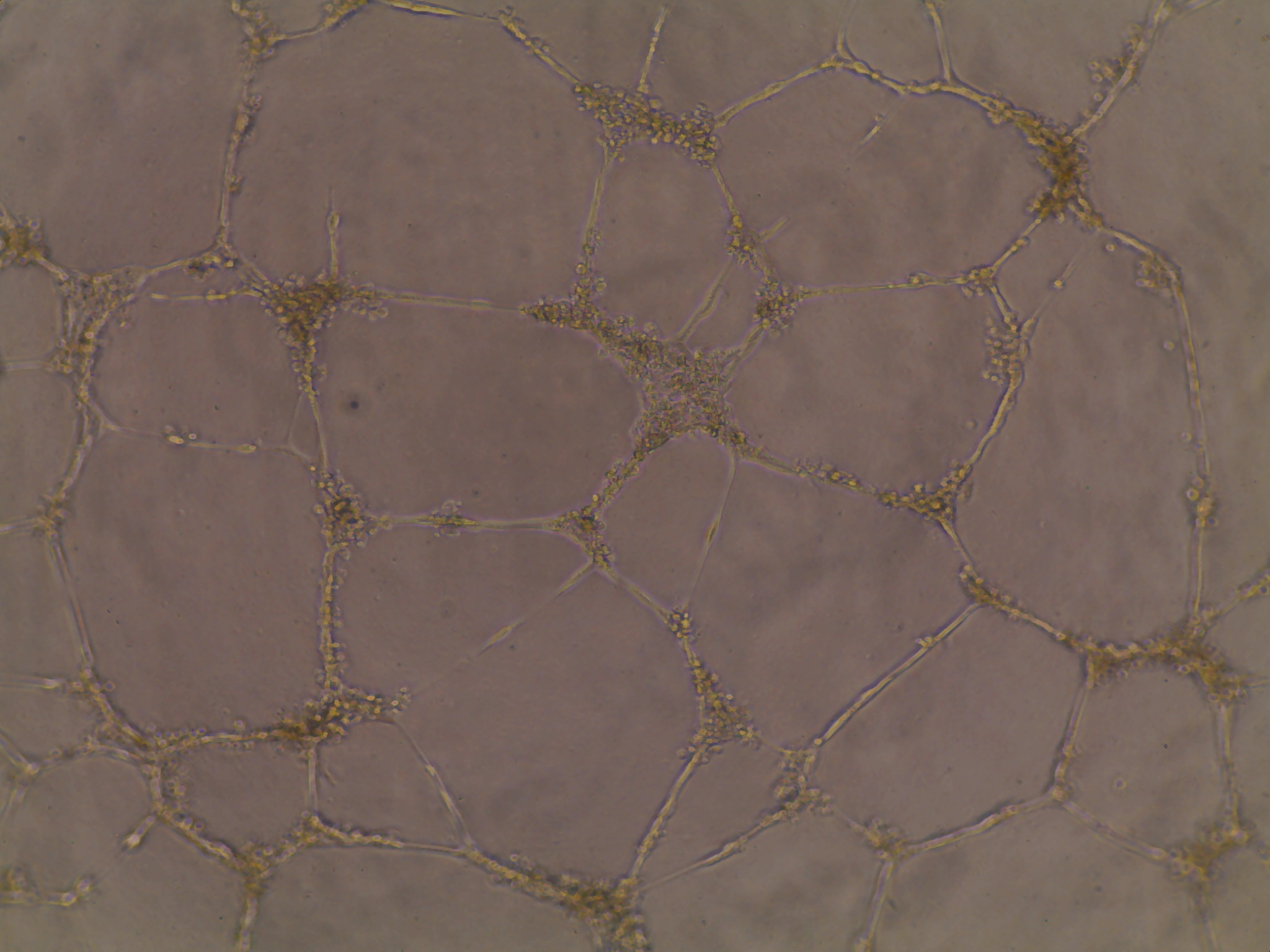

Supplement: Supplementary file 1 [file jox-15-00153-s001.zip › File S1-Original images of Figures 1a, 1b, 2a, 2c, 2e, 4a, 4c and 4e/Figure 4a/VEGF 6h.tif]

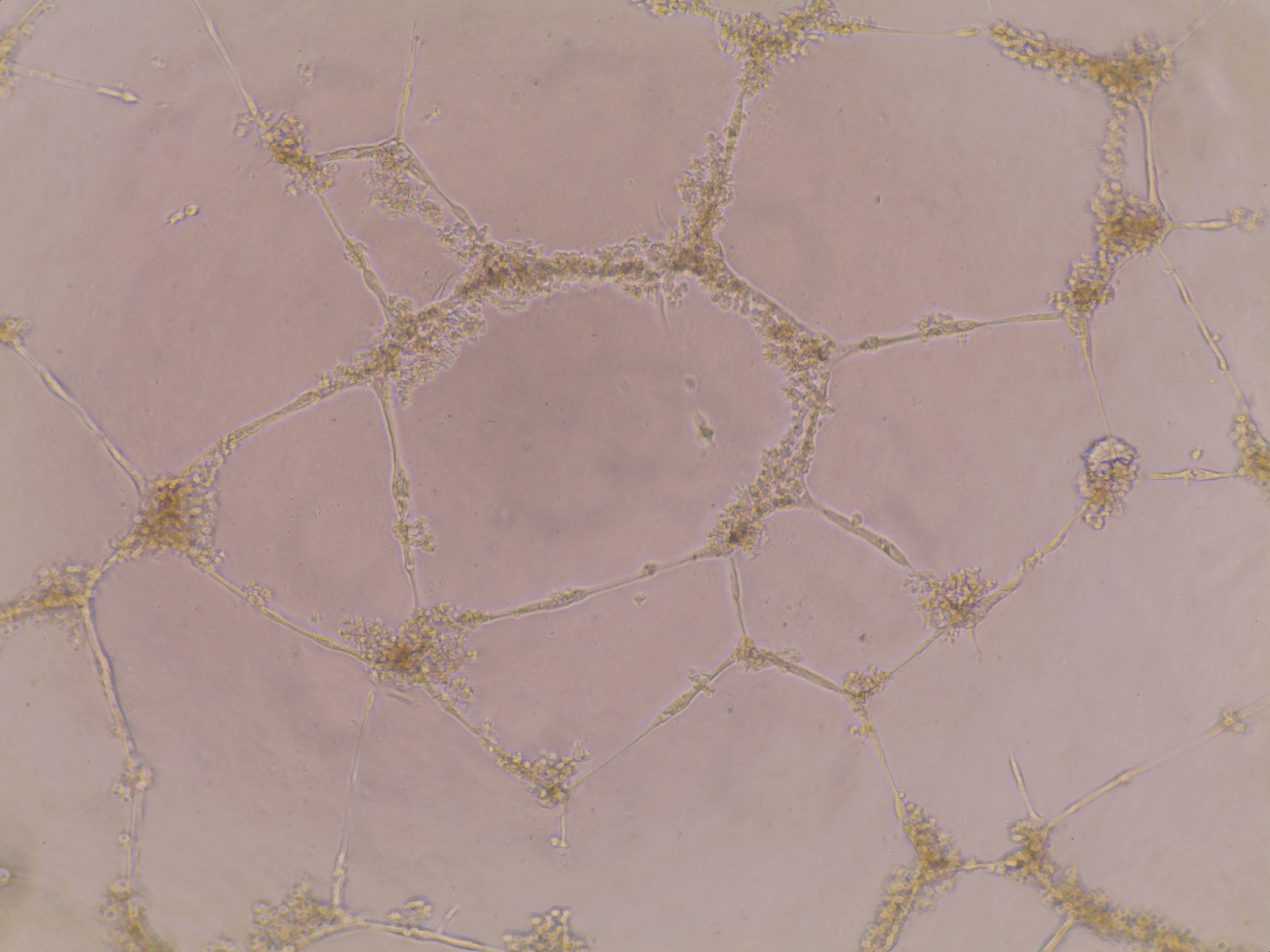

Supplement: Supplementary file 1 [file jox-15-00153-s001.zip › File S1-Original images of Figures 1a, 1b, 2a, 2c, 2e, 4a, 4c and 4e/Figure 4c/CTRL 24h.tif]

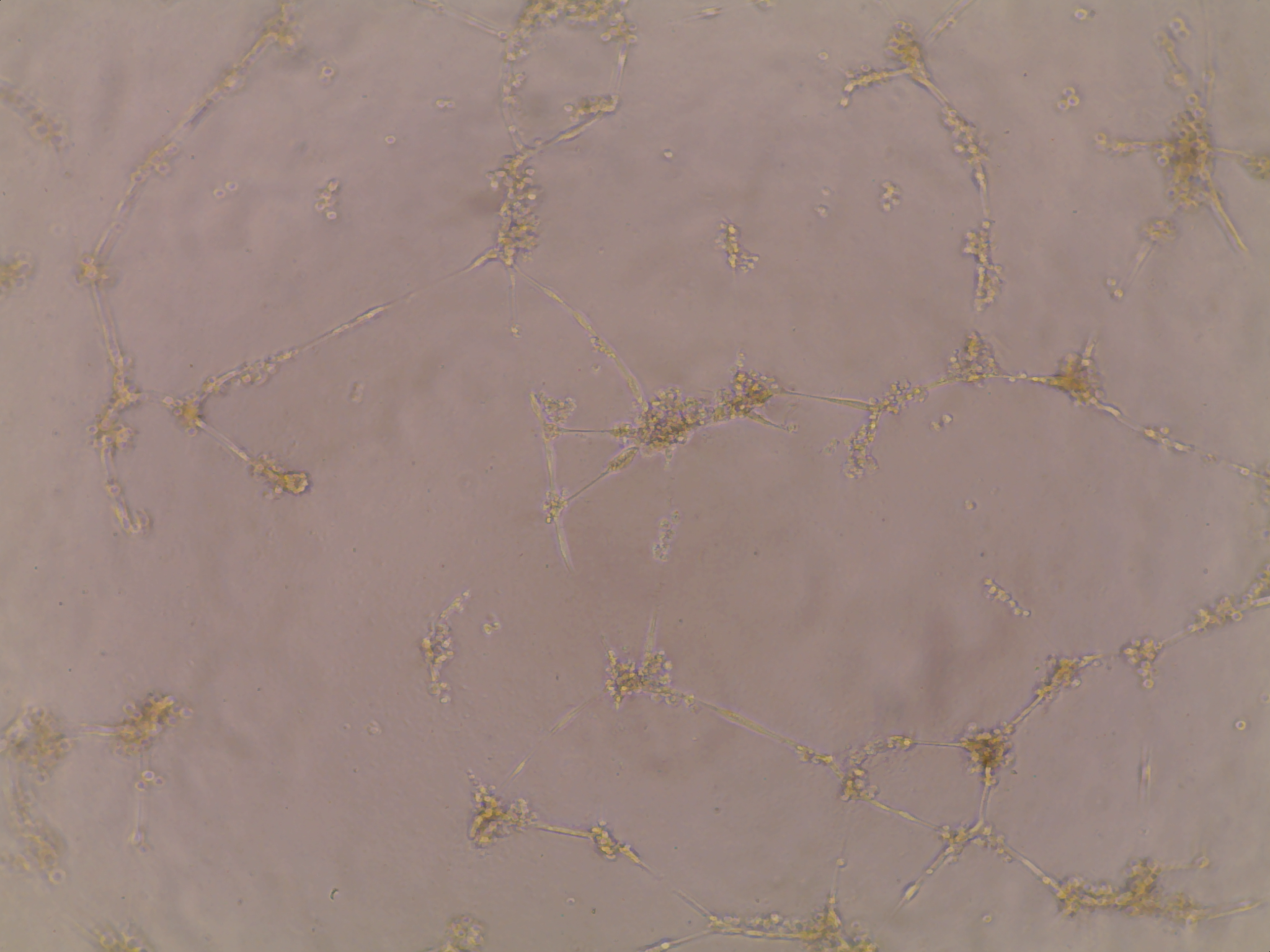

Supplement: Supplementary file 1 [file jox-15-00153-s001.zip › File S1-Original images of Figures 1a, 1b, 2a, 2c, 2e, 4a, 4c and 4e/Figure 4c/POE +VEGF 24h.tif]

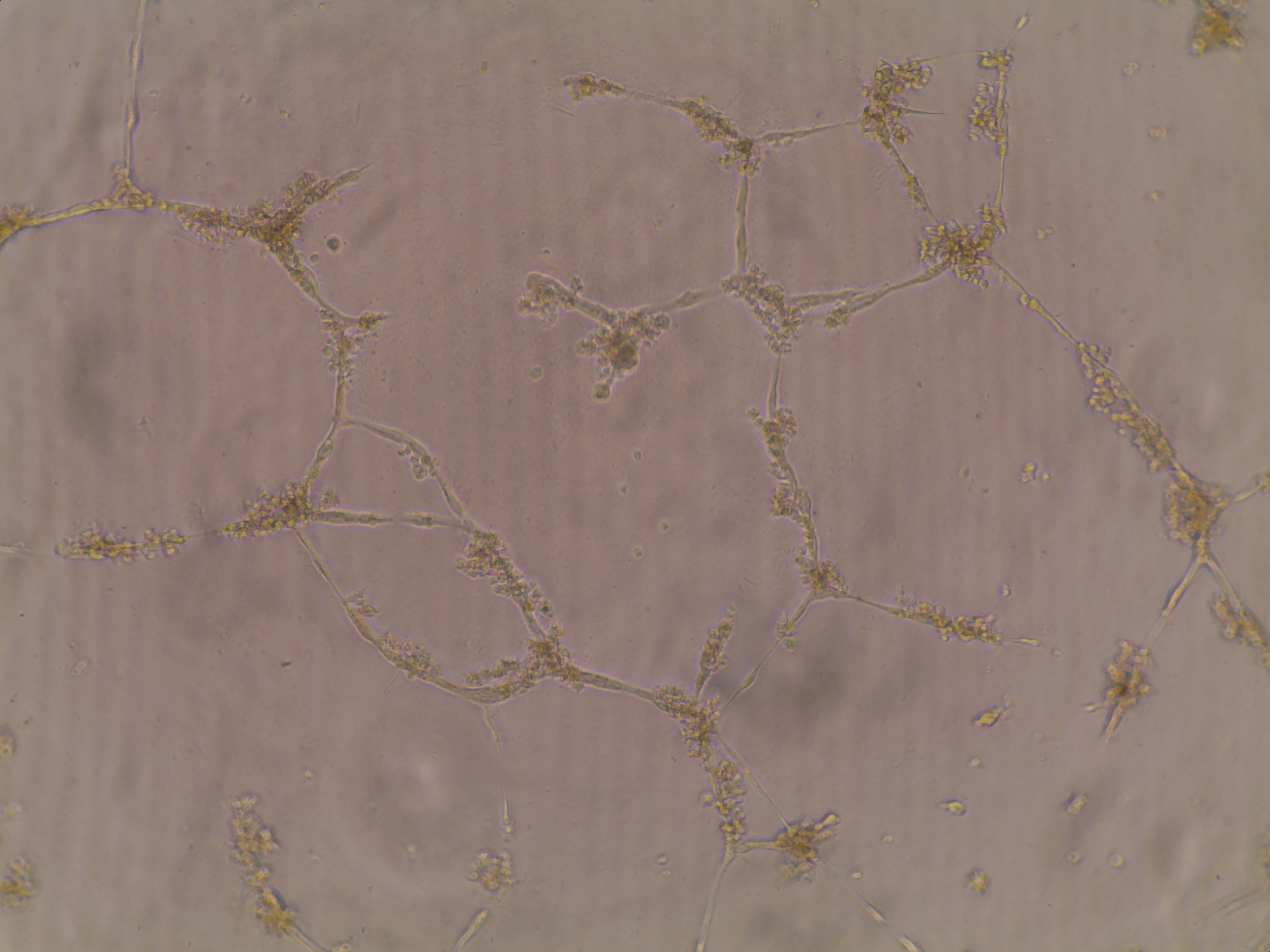

Supplement: Supplementary file 1 [file jox-15-00153-s001.zip › File S1-Original images of Figures 1a, 1b, 2a, 2c, 2e, 4a, 4c and 4e/Figure 4c/POE 24h.tif]

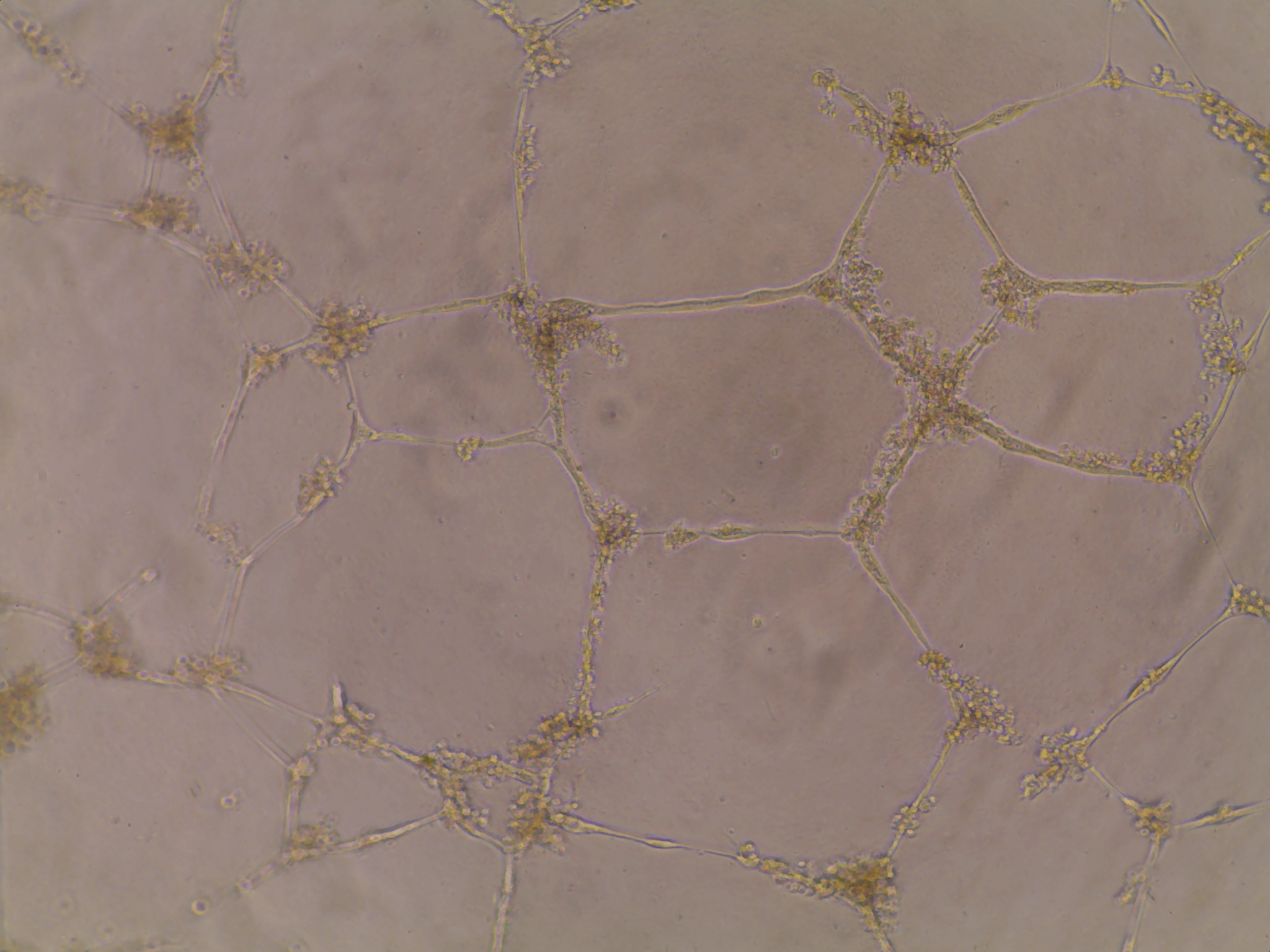

Supplement: Supplementary file 1 [file jox-15-00153-s001.zip › File S1-Original images of Figures 1a, 1b, 2a, 2c, 2e, 4a, 4c and 4e/Figure 4c/VEGF 24h.tif]

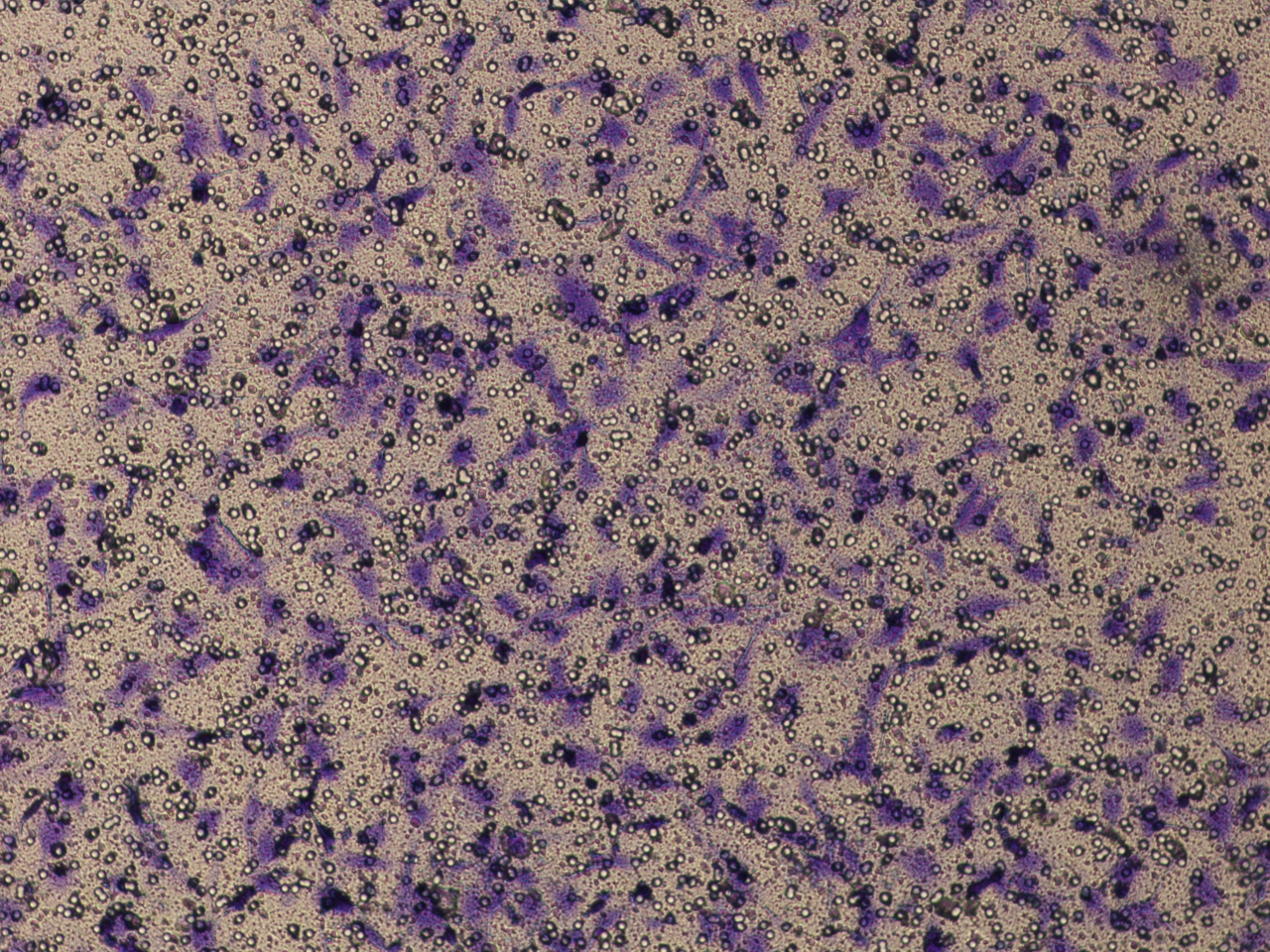

Supplement: Supplementary file 1 [file jox-15-00153-s001.zip › File S1-Original images of Figures 1a, 1b, 2a, 2c, 2e, 4a, 4c and 4e/Figure 4e/CTRL.JPG]

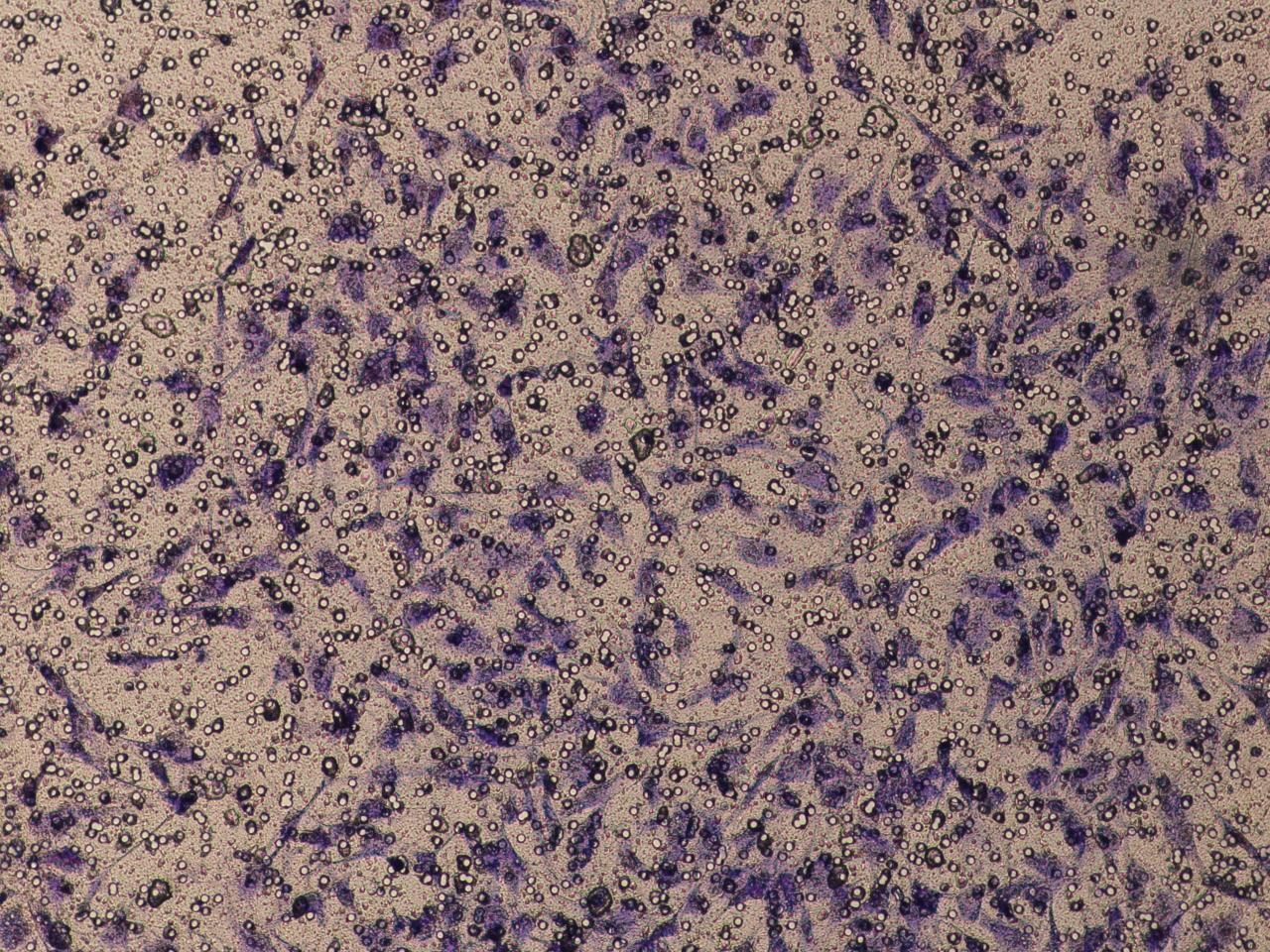

Supplement: Supplementary file 1 [file jox-15-00153-s001.zip › File S1-Original images of Figures 1a, 1b, 2a, 2c, 2e, 4a, 4c and 4e/Figure 4e/POE+VEGF.JPG]

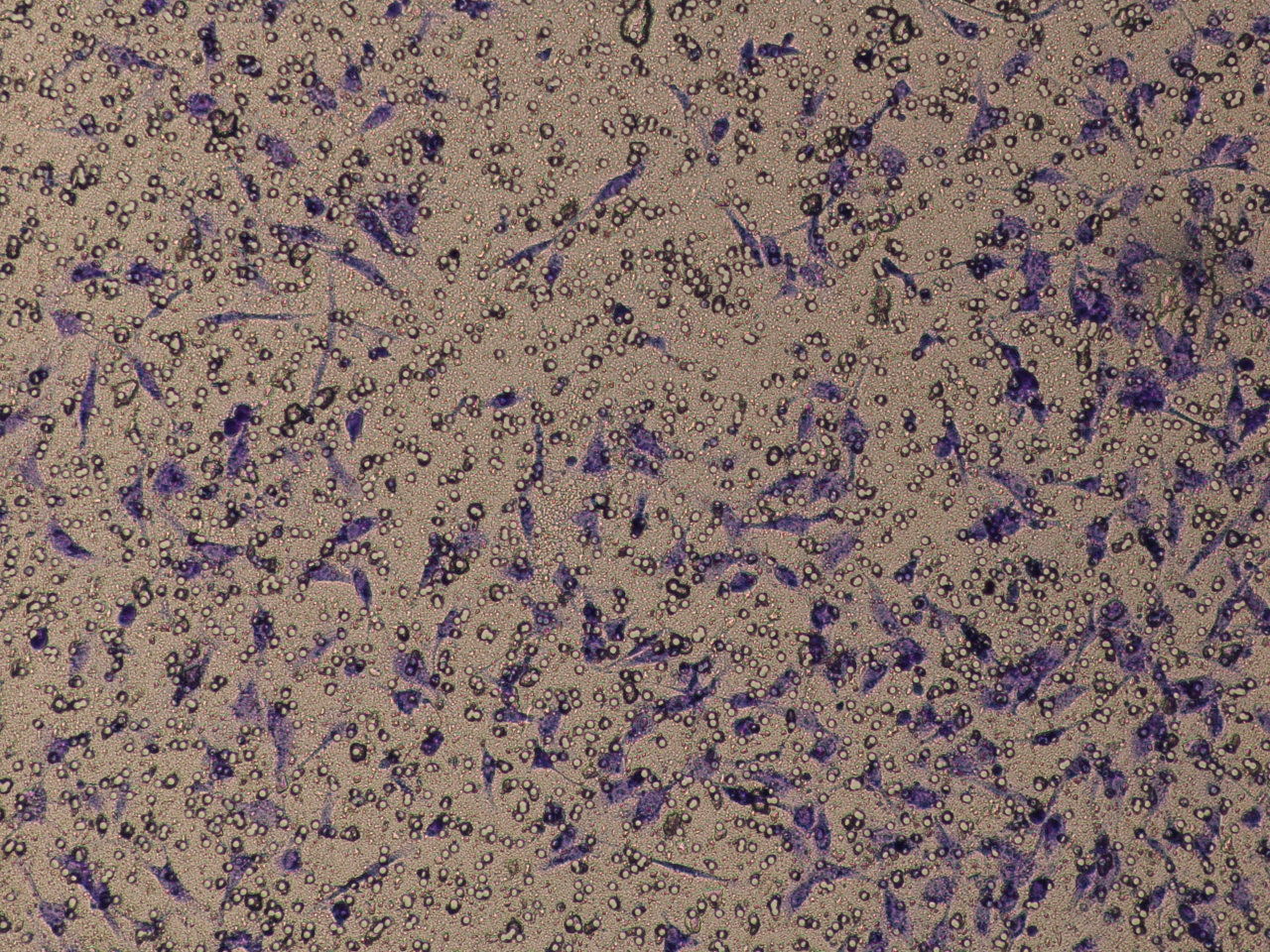

Supplement: Supplementary file 1 [file jox-15-00153-s001.zip › File S1-Original images of Figures 1a, 1b, 2a, 2c, 2e, 4a, 4c and 4e/Figure 4e/POE.JPG]

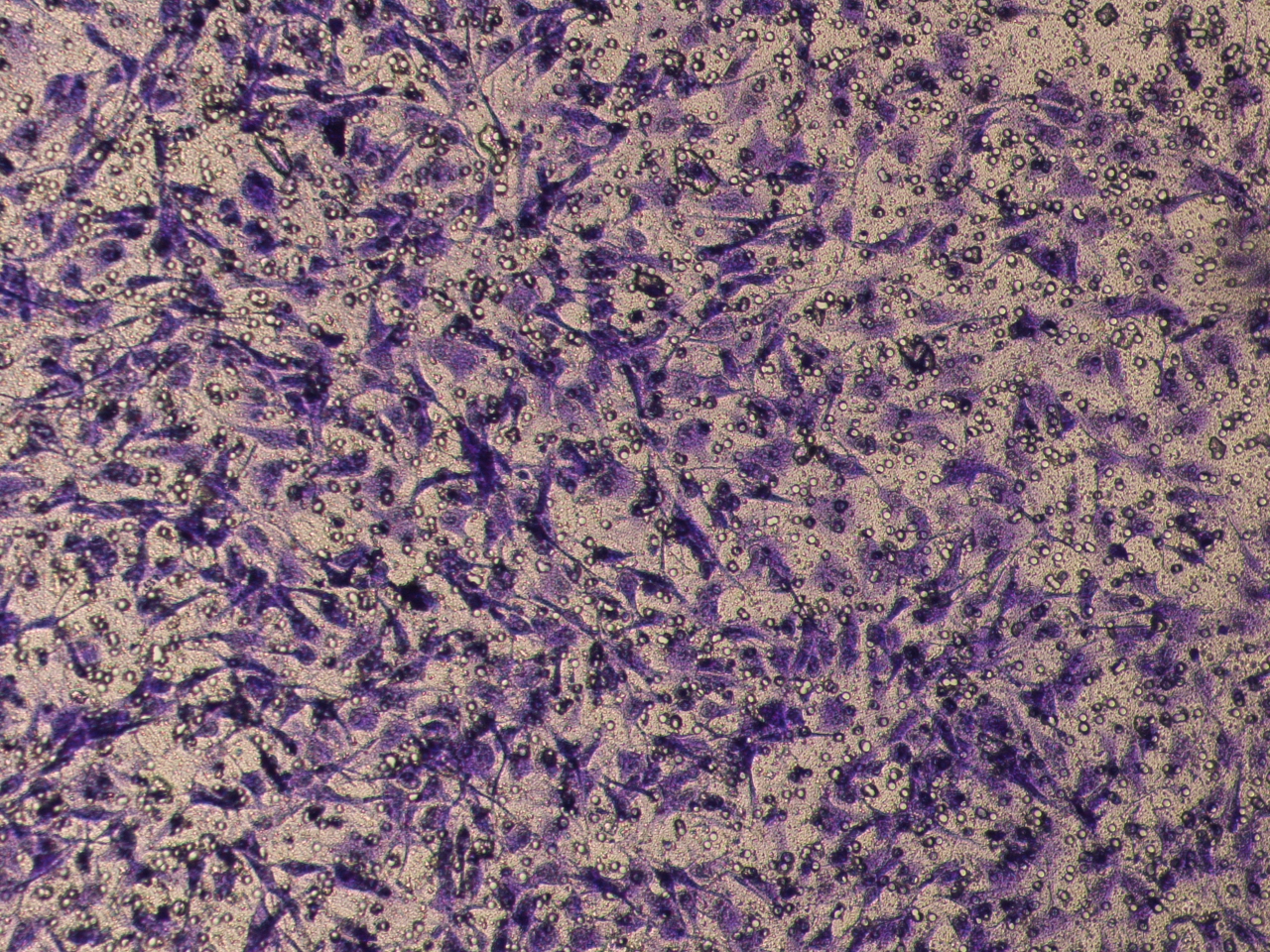

Supplement: Supplementary file 1 [file jox-15-00153-s001.zip › File S1-Original images of Figures 1a, 1b, 2a, 2c, 2e, 4a, 4c and 4e/Figure 4e/VEGF.JPG]
